# First-In-Human, Double-Blind, Placebo-Controlled, Randomized, Dose-Escalation Study of BG00010, a Glial Cell Line-Derived Neurotrophic Factor Family Member, in Subjects with Unilateral Sciatica

**DOI:** 10.1371/journal.pone.0125034

**Published:** 2015-05-11

**Authors:** Paul E. Rolan, Gilmore O’Neill, Eve Versage, Jitesh Rana, Yongqiang Tang, Gerald Galluppi, Ernesto Aycardi

**Affiliations:** 1 Clinical Pharmacology, School of Medical Sciences, University of Adelaide, Adelaide, Australia; 2 Pain and Anaesthesia Research Clinic, Royal Adelaide Hospital, Adelaide, Australia; 3 Pain Management Unit, Royal Adelaide Hospital, Adelaide, Australia; 4 Biogen IDEC, Cambridge, MA, United States of America; Griffith University, AUSTRALIA

## Abstract

**Objective:**

To evaluate the safety, tolerability, and pharmacokinetics of single doses of BG00010 (neublastin, artemin, enovin) in subjects with unilateral sciatica.

**Methods:**

This was a single-center, blinded, placebo-controlled, randomized Phase 1 sequential-cohort, dose-escalation study (ClinicalTrials.gov identifier NCT00961766; funded by Biogen Idec). Adults with unilateral sciatica were enrolled at The Royal Adelaide Hospital, Australia. Four subjects were assigned to each of eleven cohorts (intravenous BG00010 0.3, 1, 3, 10, 25, 50, 100, 200, 400, or 800 μg/kg, or subcutaneous BG00010 50 μg/kg) and were randomized 3:1 to receive a single dose of BG00010 or placebo. The primary safety and tolerability assessments were: adverse events; clinical laboratory parameters and vital signs; pain as measured by a Likert rating scale; intra-epidermal nerve fiber density; and longitudinal assessment of quantitative sensory test parameters. Blood, serum, and plasma samples were collected for pharmacokinetic and pharmacodynamic assessments. Subjects were blinded to treatment assignment throughout the study. The investigator was blinded to treatment assignment until the Data Safety Review Committee review of unblinded data, which occurred after day 28.

**Results:**

Beyond the planned enrollment of 44 subjects, four additional subjects were enrolled into to the intravenous BG00010 200 μg/kg cohort after one original subject experienced mild generalized pruritus. Therefore, a total of 48 subjects were enrolled between August 2009 and December 2011; all were included in the safety analyses. BG00010 was generally well tolerated: in primary analyses, the most common treatment-emergent adverse events were changes in temperature perception, pruritus, rash, or headache; no trends were observed in clinical laboratory parameters, vital signs, intra-epidermal nerve fiber density, or quantitative sensory testing. BG00010 was not associated with any clear, dose-dependent trends in Likert pain scores. BG00010 was rapidly distributed, with a prolonged terminal elimination phase.

**Conclusions:**

These data support the development of BG00010 for the treatment of neuropathic pain.

**Trial Registration:**

ClinicalTrials.gov NCT00961766

## Introduction

Neuropathic pain is caused by a lesion or disease of the somatosensory nervous system [1] and is associated with poor general health and a notable economic impact, resulting from increased healthcare utilization and societal costs [2]. However, effective management remains a challenge [3–6].

Recently, there has been interest in the potential use of neurotrophic factors and anti-neurotrophic factors as an approach to pain modification. BG00010 (neublastin, artemin, enovin; Biogen Idec Inc., Cambridge, MA, USA) is a member of the glial cell line-derived neurotrophic factor family of ligands [7]. In cell cultures, BG00010 has been found to activate downstream signaling through the rearranged-during-transfection (RET) receptor tyrosine kinase by binding to the GFRα3 co-receptor [8], which is principally expressed within small-diameter dorsal root ganglion cells, with increased expression following axonal damage [9]. In terms of function, BG00010 has been found to promote the survival of sensory neurons from the dorsal root ganglion, as well as other sensory, sympathetic, and central neurons [8].

Subsequent studies have indicated that BG00010 may have a protective role in whole animals: it has been found to prevent histochemical changes to dorsal root ganglion cells, maintain C-fiber function, and restore sensory neuronal function following nerve injury in adult rats [10,11].

BG00010 has demonstrated efficacy in preclinical models of neuropathic pain. A 113-amino acid form of rat BG00010 derived from *Escherichia coli* provided sustained improvements in neuropathic pain produced by ligation of the L5 (sciatic) and L6 spinal nerves in rats, without producing sensory or motor abnormalities [12]. In a similar rat model produced by chronic constriction of the L5 nerve, time- and dose-dependent alleviation of neuropathic pain was achieved following intravenous (i.v.) administration of a human recombinant version of BG00010, derived from transfected Chinese hamster ovary cells [13,14]. The mechanism of action remains unclear, but it is thought that BG00010 may selectively normalize the pathophysiological mechanisms that mediate pain, without impacting on sensory or motor functioning.

Here we report the first-in-human administration of BG00010 in subjects with unilateral sciatica in a randomized, blinded, placebo-controlled, Phase 1 dose-escalation study. The primary objective of the study was to determine the safety, tolerability, systemic pharmacokinetic behavior, and immunogenicity of single injections of BG00010 via i.v. (0.3–800 μg/kg) or subcutaneous (s.c.; 50 μg/kg) administrations.

## Methods

The protocol for this trial and supporting CONSORT checklist are available as S1 Checklist and S1 Protocol.

### Study Design

This was a single-center, blinded, placebo-controlled, randomized Phase 1 sequential-cohort, dose-escalation study to evaluate the safety, tolerability, and pharmacokinetic profile of BG00010 after i.v. or s.c. administration of a single dose in subjects with sciatica. The study was conducted at the Pain and Anaesthesia Research Clinic, Royal Adelaide Hospital, Adelaide, Australia, and was registered with ClinicalTrials.gov (NCT00961766). The study protocol and amendments were reviewed and approved by the Research Ethics Committee of the hospital. The study was performed in accordance with the Declaration of Helsinki, the US Code of Federal Regulations, all relevant European Directives, and the International Conference on Harmonisation Guideline on Good Clinical Practice. All subjects gave written informed consent before trial participation.

### Subjects

Eligible subjects were recruited from the community by advertisement. Screening to assess subject eligibility and seek informed consent took place within 21 days prior to the baseline visit. Subjects were aged 18 to 70 years and had a clinical diagnosis of unilateral sciatica with symptoms that had been present for at least 6 weeks before screening and a pain rating of ≥40 mm on the 100 mm visual analog scale (VAS) of the Short-Form McGill Pain Questionnaire (SF-MPQ) at screening and baseline. All participants were screened by an accredited pain physician, with the diagnosis made on the basis of a history of pain and a dermatomal distribution in the lower leg. Neuropathic components such as allodynia, paresthesia, sensory loss and areflexia/hyporeflexia in the dermatome and MRI/CT findings of nerve route impingement consistent with the dermatomal distribution of symptoms were regarded as confirmatory but were not required for inclusion into the study. Key exclusion criteria included a history of signs or symptoms of peripheral neuropathy other than symptoms of sciatica, and the presence of an active pain condition with intensity similar to or worse than that of sciatica. Concomitant treatment with analgesics and/or pain-modifying drugs was permitted if doses had been stabilized before the baseline visit. Doses of selective serotonin reuptake inhibitors, serotonin noradrenaline reuptake inhibitors, and tricyclic antidepressants must have been stable for 4 weeks prior to baseline. Gabapentin and pregabalin doses must have been stable for at least 1 week prior to baseline.

### Treatment

It was planned that four subjects would be assigned to each of eleven cohorts: BG00010 i.v. infusion 0.3, 1, 3, 10, 25, 50, 100, 200, 400, or 800 μg/kg, or BG00010 s.c. 50 μg/kg. The length of the i.v. infusions varied from 3 min at the lower doses to 12 min at the higher doses. The s.c. cohort was added as part of a protocol amendment to explore the feasibility of s.c. administration. If required, four additional subjects could be added to a cohort after data review by the Data Safety Review Committee. Within each i.v. and s.c. cohort, once eligibility had been confirmed by the investigator, subjects were formally enrolled, assigned a subject identification number and randomized 3:1 to receive BG00010 or placebo (saline). The computer-generated master randomization list was prepared before the start of the study and given to an unblinded pharmacist.

The doses and dosing schedule were based on previous non-clinical toxicology results and the projected efficacious human dose of BG00010; the s.c. dose level was restricted to 50 μg/kg due to formulation limitations. The sample size of this exploratory study was based on previous classical study designs and was not based on any study power considerations.

Only one subject received treatment on any study day. Subjects entered the inpatient unit within 72 h before administration of treatment (baseline, day -1). Subjects were monitored in the inpatient unit for at least 48 h following treatment (days 0, 1, and 2), and returned to the clinic for follow-up visits on days 3, 5, 7, 21, 28, and 56. The Data Safety Review Committee reviewed unblinded safety and pharmacokinetic data for each i.v. cohort before enrollment of the cohort at the next planned dose level. Enrollment for the s.c. cohort took place after completion of dosing of the i.v. cohorts. The overall duration of participation for each subject was approximately 11 weeks.

Subjects were blinded to treatment assignment throughout the study. The investigator was blinded to treatment assignment until the Data Safety Review Committee review of unblinded data, which occurred after day 28.

### Safety and Pain Assessments

The primary safety and tolerability endpoints were: number and proportion of subjects with adverse events (AEs; coded using the Medical Dictionary for Regulatory Activities version 14); clinical laboratory parameters and vital signs; pain as measured by a Likert numerical pain rating scale (assessed within 7 days of baseline, 30 min before treatment, and after treatment at 15 and 45 min, 1, 4, 6, 9, and 12 h, and days 1, 2, 3, 5, 7, 21, 28, and 56; day 56 assessments only included for subjects in BG00010 i.v. 100–800 μg/kg or BG00010 s.c. 50 μg/kg cohorts); intra-epidermal nerve fiber density (IENFD; determined using two punch biopsies taken from the non-sciatica-affected leg between screening and baseline and at day 28, mean reductions from screening of ≥2 standard deviations [SDs] flagged; not evaluated for subjects treated with BG00010 s.c. 50 μg/kg); and longitudinal assessment of five quantitative sensory test (QST) parameters: vibratory sensation, cool thermal, and heat pain thresholds, and cool thermal and heat pain tolerances (means of duplicate measurements recorded at screening, baseline, day 1, and day 28; mean reductions from baseline of ≥2 SDs flagged; not evaluated for subjects treated with BG00010 s.c. 50 μg/kg).

A subject was considered to have a protocol-defined worsening of sensory function if two or more of the following events were present: (1) worsening of vibratory sensation from baseline on clinical neurological examination (from normal to reduced, from normal to absent at root, or from reduced to absent at root); (2) change from baseline in average QST of ≥2 SDs for any of the five QST parameters; (3) reduction from baseline in IENFD of ≥2 SDs.

Subjects also completed the VAS of the SF-MPQ at screening, baseline, days 28 and 56, and the end of study/premature study withdrawal visit (day 56 assessments only included for subjects in BG00010 i.v. 100–800 μg/kg or BG00010 s.c. 50 μg/kg cohorts). The type of neuropathic pain assessed could include both back and leg pain. All subjects who were randomized and treated were included in these safety analyses.

IENFD, QST and SF-MPQ data were analyzed using Statistical Analysis Software, version 9.2.

In addition, incidences of BG00010-binding and-neutralizing antibodies were summarized for all subjects who were randomized, treated, and had post-dose immunogenicity data.

### Pharmacokinetic and Pharmacodynamic Assessments

Blood samples were collected from each subject in the i.v. cohorts at 30 min before treatment, and after treatment at 15 min and 30 min, and 1, 2, 3, 4, 6, 9, 12, 18, 24, 48, 72, and 120 h. The same blood sampling schedule was used in the s.c. cohort, except that no sample was taken at 15 min after treatment.

Serum concentrations of BG00010 were measured using a chemiluminescent enzyme-linked immunosorbent assay. The lower limit of quantitation for BG00010 was 0.100 ng/ml; serum concentrations below the limit of quantitation were set equal to 0 ng/ml. Pharmacokinetic analysis of BG00010 serum concentration versus time data was performed using the non-compartmental analysis function of WinNonlin Phoenix, version 6.1.

The following primary pharmacokinetic endpoints for the i.v. cohorts were analyzed: maximum observed serum concentration (C_max_), area under the serum concentration—time curve from time zero to infinity (AUC_inf_), terminal half-life (t_½_), total body clearance (Cl), and volume of distribution at steady state (V_ss_). For the s.c. cohort, time to C_max_ (T_max_) was also assessed, as well as bioavailability. All subjects who were randomized, treated, and had measurable BG00010 serum concentrations from at least one collected sample were included in the pharmacokinetic analyses.

For pharmacodynamic analyses, blood, serum, and plasma samples were collected for laboratory assessments of substance P, monocyte chemoattractant protein-1, and norepinephrine at baseline and after treatment at 15 min, 1, 6, 24, and 72 h, and 28 days. All subjects who were randomized, treated, and had post-dose samples analyzed for biomarkers were included in the pharmacodynamic analyses. Pharmacodynamic data were analyzed using Statistical Analysis Software, version 9.2.

## Results

### Subjects

**Fig 1:**
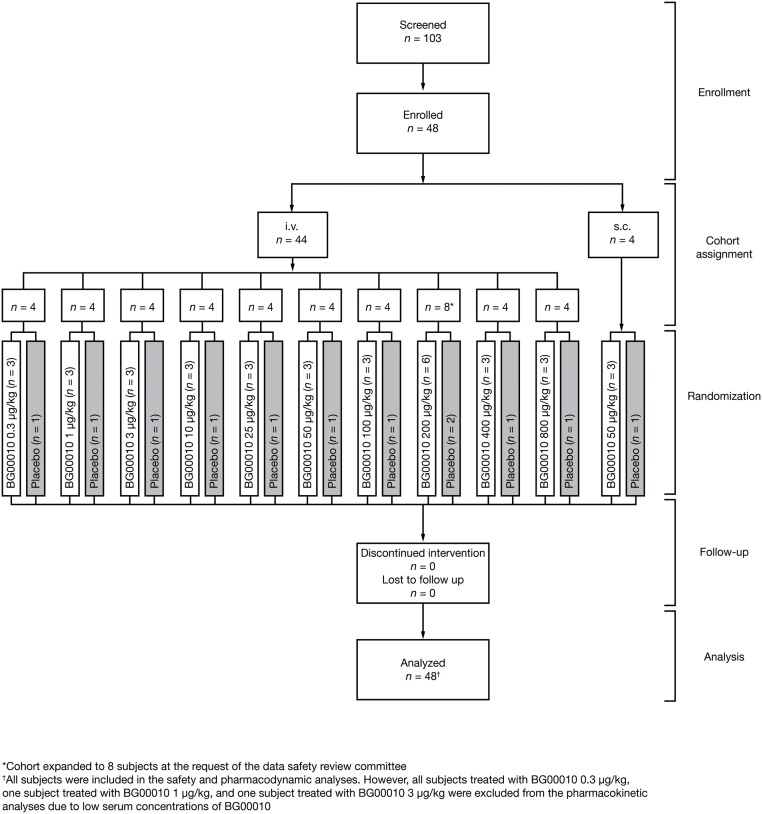
Subject disposition. i.v., intravenous; s.c., subcutaneous.

Of 545 subjects included in a preliminary telephone screening stage, 103 completed a full screen, of whom 46 failed, and a further nine passed but did not enter the study. The remaining 48 subjects were enrolled between August 31, 2009 and December 13, 2011, and randomized to study treatment (Fig 1): beyond the planned enrollment of 44 subjects, the Data Safety Review Committee required the enrollment of four additional subjects to the BG00010 i.v. 200 μg/kg cohort after one original subject in this cohort experienced mild generalized pruritus that lasted >30 days. All 48 subjects were included in the safety analyses.

Baseline demographics and clinical characteristics are shown in Table 1. Subjects’ age ranged from 23 to 70 years; 47/48 (97.9%) subjects were white and one was Asian. Most subjects (43/48; 89.6%) were receiving concomitant pain medication, the most common being acetaminophen (20/48; 41.7%) and a combination of acetaminophen and codeine phosphate (11/48; 22.9%).

**Table 1:** Baseline demographics and clinical characteristics.

	BG00010												Placebo
	i.v.										s.c.	Total	
**Dose, μg/kg**	**0.3**	**1**	**3**	**10**	**25**	**50**	**100**	**200**	**400**	**800**	**50**	**N/A**	**N/A**
No. of subjects	3	3	3	3	3	3	3	6	3	3	3	36	12
Age, years, mean (SD)	50.7 (9.1)	48.3 (22.0)	49.0 (6.6)	55.0 (11.1)	45.7 (8.1)	55.7 (16.3)	56.3 (4.0)	55.0 (10.6)	54.7 (9.5)	43.0 (14.0)	58.7 (5.9)	52.3 (10.9)	61.5 (8.1)
Male, *n*	1	1	0	2	2	2	1	2	2	2	2	17	5
White, *n*	3	2	3	3	3	3	3	6	3	3	3	35	12
BMI, kg/m^2^, mean (SD)	25.8 (4.9)	25.2 (5.5)	29.6 (1.9)	27.3 (1.0)	28.6 (2.3)	28.9 (1.9)	27.8 (1.9)	27.3 (2.6)	26.8 (5.2)	25.7 (2.9)	28.8 (2.2)	27.4 (3.0)	28.0 (2.8)
SF-MPQ VAS score, mean (SD)	75.3 (9.3)	47.7 (7.2)	59.3 (22.0)	49.0 (10.6)	66.7 (8.5)	61.7 (13.0)	65.0 (17.1)	68.5 (26.9)	43.7 (2.1)	69.0 (24.4)	56.3 (11.0)	60.9 (17.6)	62.3 (14.6)
*Receiving medication at screening*, *n*													
Pain medication	3	1	2	2	3	2	3	6	3	3	3	31	12
Any other medication	2	1	3	2	2	2	3	5	2	1	1	24	12

BMI, body mass index; i.v., intravenous; N/A, not applicable; s.c., subcutaneous; SD, standard deviation; SF-MPQ, Short-Form McGill Pain Questionnaire; VAS, visual analog scale.

### Adverse Events

Treatment-emergent adverse events (TEAEs) affected 100.0% of placebo- and BG00010-treated subjects. The most commonly reported TEAEs with placebo were headache (25%) and dizziness (25%; Table 2). Four subjects receiving placebo experienced severe TEAEs (worsening of sciatica, *n* = 2; back pain, *n* = 1; foot fracture, *n* = 1; procedural pain, *n* = 1). With BG00010 (any dose), the most commonly reported TEAEs were feeling hot (39%), pruritus (39%), headache (36%), pruritus generalized (25%), and rash (25%; Table 2). In general, the incidences of feeling hot, pruritus, and pruritus generalized were higher at BG00010 doses of ≥100 μg/kg. Two subjects receiving BG00010 experienced severe TEAEs (nausea and flare-up of pre-existing diverticulitis, *n* = 1 [i.v. 3 μg/kg]; worsening of sciatica, *n* = 1 [s.c. 50 μg/kg]).

**Table 2:** Treatment-emergent adverse events.

	BG00010												Placebo
	i.v.										s.c.	Total	
**Dose, μg/kg**	**0.3**	**1**	**3**	**10**	**25**	**50**	**100**	**200**	**400**	**800**	**50**	**N/A**	**N/A**
No. of subjects	3	3	3	3	3	3	3	6	3	3	3	36	12
Feeling hot, *n* (%)	0 (0.0)	0 (0.0)	0 (0.0)	1 (33.3)	0 (0.0)	1 (33.3)	2 (66.7)	4 (66.7)	3 (100.0)	3 (100.0)	0 (0.0)	14 (38.9)	2 (16.7)
Pruritus, *n* (%)	0 (0.0)	0 (0.0)	0 (0.0)	1 (33.3)	0 (0.0)	1 (33.3)	3 (100.0)	4 (66.7)	3 (100.0)	1 (33.3)	1 (33.3)	14 (38.9)	2 (16.7)
Headache, *n* (%)	3 (100.0)	1 (33.3)	2 (66.7)	1 (33.3)	1 (33.3)	0 (0.0)	2 (66.7)	1 (16.7)	1 (33.3)	1 (33.3)	0 (0.0)	13 (36.1)	3 (25.0)
Pruritus generalized, *n* (%)	0 (0.0)	0 (0.0)	0 (0.0)	0 (0.0)	0 (0.0)	2 (66.7)	0 (0.0)	4 (66.7)	1 (33.3)	1 (33.3)	1 (33.3)	9 (25.0)	0 (0.0)
Rash, *n* (%)	0 (0.0)	0 (0.0)	0 (0.0)	0 (0.0)	0 (0.0)	2 (66.7)	3 (100.0)	2 (33.3)	1 (33.3)	1 (33.3)	0 (0.0)	9 (25.0)	0 (0.0)
Pain in extremity, *n* (%)	1 (33.3)	2 (66.7)	0 (0.0)	1 (33.3)	0 (0.0)	0 (0.0)	0 (0.0)	1 (16.7)	0 (0.0)	0 (0.0)	0 (0.0)	5 (13.9)	2 (16.7)
Worsening of sciatica, *n* (%)	0 (0.0)	0 (0.0)	0 (0.0)	0 (0.0)	0 (0.0)	0 (0.0)	1 (33.3)	1 (16.7)	1 (33.3)	1 (33.3)	1 (33.3)	5 (13.9)	2 (16.7)
Fatigue, *n* (%)	0 (0.0)	0 (0.0)	1 (33.3)	0 (0.0)	1 (33.3)	0 (0.0)	0 (0.0)	0 (0.0)	1 (33.3)	0 (0.0)	0 (0.0)	3 (8.3)	2 (16.7)
Dizziness, *n* (%)	0 (0.0)	0 (0.0)	1 (33.3)	0 (0.0)	0 (0.0)	0 (0.0)	1 (33.3)	0 (0.0)	0 (0.0)	0 (0.0)	0 (0.0)	2 (5.6)	3 (25.0)

Treatment-emergent adverse events affecting ≥15% of subjects treated with BG00010 (any dose) or ≥15% of subjects treated with placebo.

i.v., intravenous; N/A, not applicable; s.c., subcutaneous.

**Fig 2:**
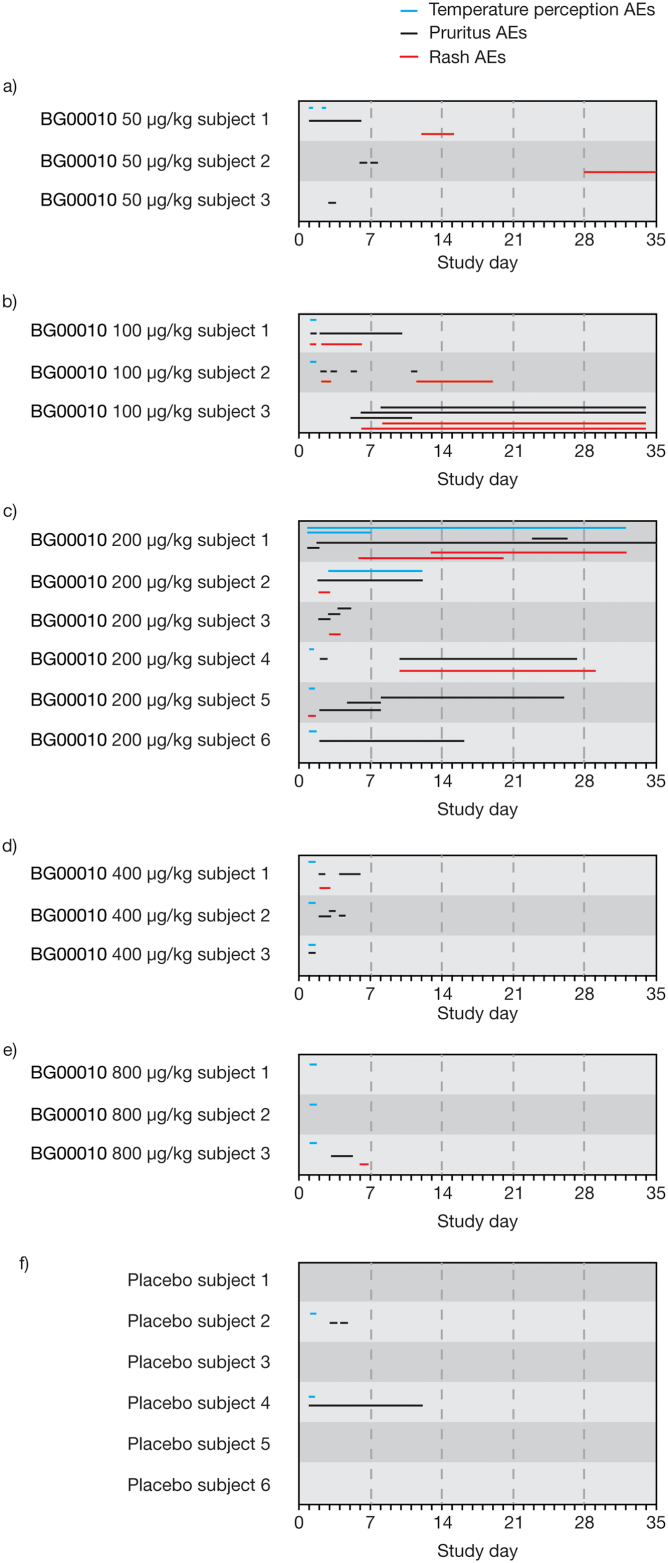
Incidence and duration of temperature perception, pruritus and rash AEs. Subjects were treated as follows: intravenous BG00010 (a) 50 μg/kg, (b) 100 μg/kg, (c) 200 μg/kg, (d) 400 μg/kg, (e) 800 μg/kg, or (f) placebo. AE, adverse event.

At doses of BG00010 ≥50 μg/ml, there were three groups of AEs that were most frequently observed: temperature perception AEs (feeling hot, hot flush, temperature intolerance, burning sensation, feeling cold), pruritus AEs, and rash AEs. Fig 2 shows the occurrences and time courses of these groups of AEs in subjects receiving BG00010 i.v. 50–800 μg/kg. There were broad variations in combinations, timings, and durations (temperature perception AEs, up to 32 days; pruritus AEs, up to 34 days; rash AEs, up to 29 days), with no clear dose-related trends for frequency or severity.

Treatment-related AEs were reported by 33.3% of subjects treated with placebo and 72.2% of subjects treated with BG00010. All of these AEs resolved by the end of follow-up, with only two exceptions in subjects receiving BG00010: mild pain in extremity (*n* = 1 [i.v. 1 μg/kg]) and mild sensory disturbance (*n* = 1 [i.v. 1 μg/kg]).

Two serious AEs were reported during the study (foot fracture, *n* = 1 [placebo]; diverticulitis, *n* = 1 [BG00010 i.v. 3 μg/kg]; both graded severe, as detailed above), neither of which was considered related to treatment. There were no deaths and no subjects withdrew from the study due to AEs.

### Clinical Laboratory Parameters and Vital Signs

There were no clinically significant changes in hematology or urinalysis parameters, vital signs, physical examination findings, electrocardiogram results, or clinical neurological examination findings. In terms of blood chemistry, there were two subjects with increased serum lipase levels (BG00010 i.v. 25 μg/kg, *n* = 1; placebo, *n* = 1) that resulted in a Common Terminology Criteria for Adverse Events (CTCAE; Version 3.0) rating of grade 3 (2.0–5.0 times the upper limit of normal) at day 2, but both resolved by day 5 (although one subject treated with BG00010 i.v. 25 μg/kg experienced recurrence at day 28). In addition, another subject experienced increased aspartate transaminase/serum glutamic oxaloacetic transaminase and lactate dehydrogenase levels (BG00010 i.v. 800 μg/kg, *n* = 1), which was considered to be an AE, but did not result in a CTCAE rating of grade 3. None of these blood chemistry results was considered to be related to treatment.

### Pain

**Fig 3:**
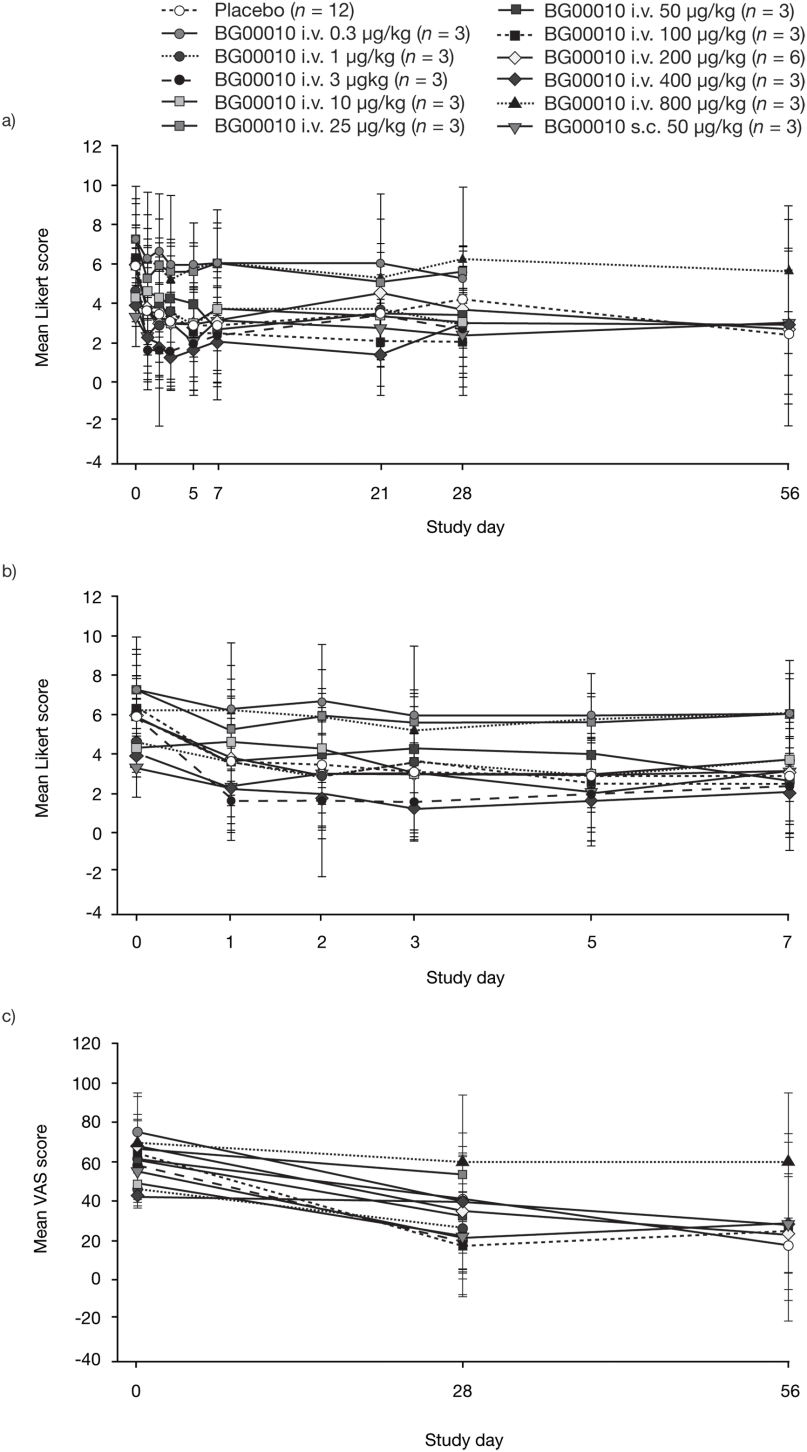
Pain outcomes over time. Mean (standard deviation) scores on the Likert numerical pain scale over (a) 56 days and (b) 7 days (expanded time axis), and (c) mean (standard deviation) scores on the 100 mm VAS of the Short-Form McGill Pain Questionnaire over 56 days, following i.v. or s.c. administration of BG00010 or placebo. Note that Likert data were missing at day 21 for one placebo-treated subject, and both Likert and McGill data were missing at day 56 for six placebo-treated subjects and all subjects treated with BG00010 0.3, 1, 3, 10, 25 or 50 μg/kg. i.v., intravenous; s.c., subcutaneous; VAS, visual analog scale.

Between baseline and post-treatment timepoints, BG00010 was not associated with any clear, dose-dependent trends in Likert numerical pain scores or scores on the VAS of the SF-MPQ (Fig 3).

### Intra-Epidermal Nerve Fiber Density

**Fig 4:**
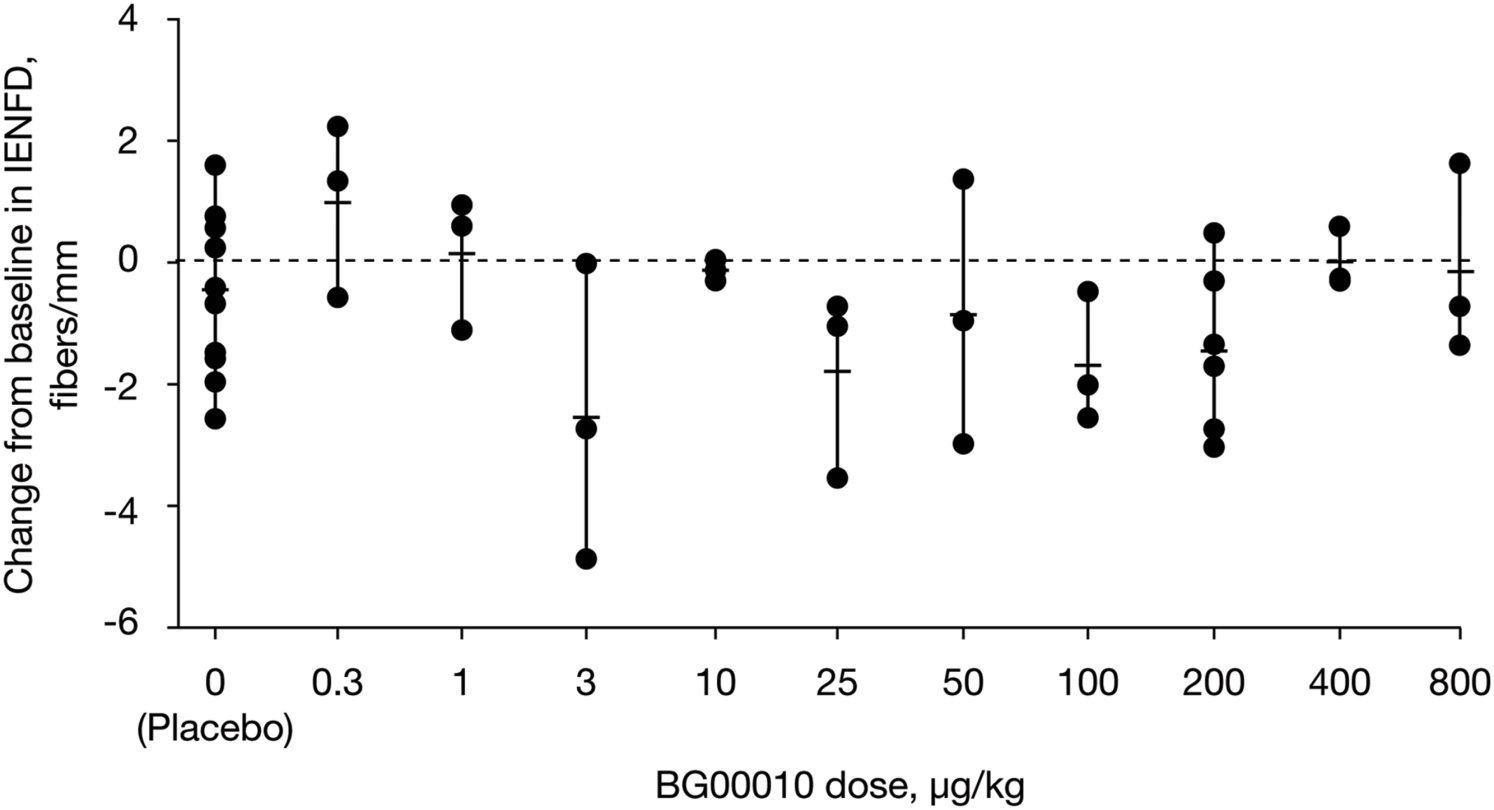
Change in IEFND between baseline and day 28. Data points indicate data for individual subjects following intravenous administration of BG00010 or placebo; lines indicate mean and range. IEFND, intra-epidermal nerve fiber density.

In general, variability of IENFD was within the expected range, with no dose-dependent trends and most day 28 readings within plus or minus two nerve fibers of baseline readings (Fig 4). Changes from baseline of ≥2 SDs in QST parameters were observed in two subjects treated with placebo: one subject had a reduced cool thermal threshold at day 1 that resolved by day 28, and one subject had an increased heat pain threshold at day 28 only. In subjects treated with BG00010, there were four subjects with reductions in cool thermal threshold of ≥2 SDs (day 1 only [resolved by day 28], *n* = 2; day 1 and day 28, *n* = 1; day 28 only, *n* = 1), one subject with a reduction in cool thermal tolerance of ≥2 SDs (day 28 only), two subjects with increases in cool thermal tolerance of ≥2 SDs (day 1 and day 28, *n* = 1; day 28 only, *n* = 1), one subject with increases in both cool thermal threshold and tolerance of ≥2 SDs (day 28 only), and one subject with a reduction in heat pain threshold of ≥2 SDs (day 1 only [resolved by day 28]). There were no overall trends for increases or decreases in QST parameters from baseline. One subject treated with placebo experienced a worsening in sensory function as indicated by a worsening of vibratory sensation from baseline based on clinical neurological examination and a change in cool thermal threshold of ≥2 SDs from baseline.

### Immunogenicity

Although BG00010-binding antibodies were detected in two subjects (BG00010 i.v. 50 μg/kg, *n* = 1; BG00010 s.c. 50 μg/kg, *n* = 1), both had negative results in a subsequent assay for neutralizing antibodies.

### Pharmacokinetic and Pharmacodynamic Assessments

**Fig 5:**
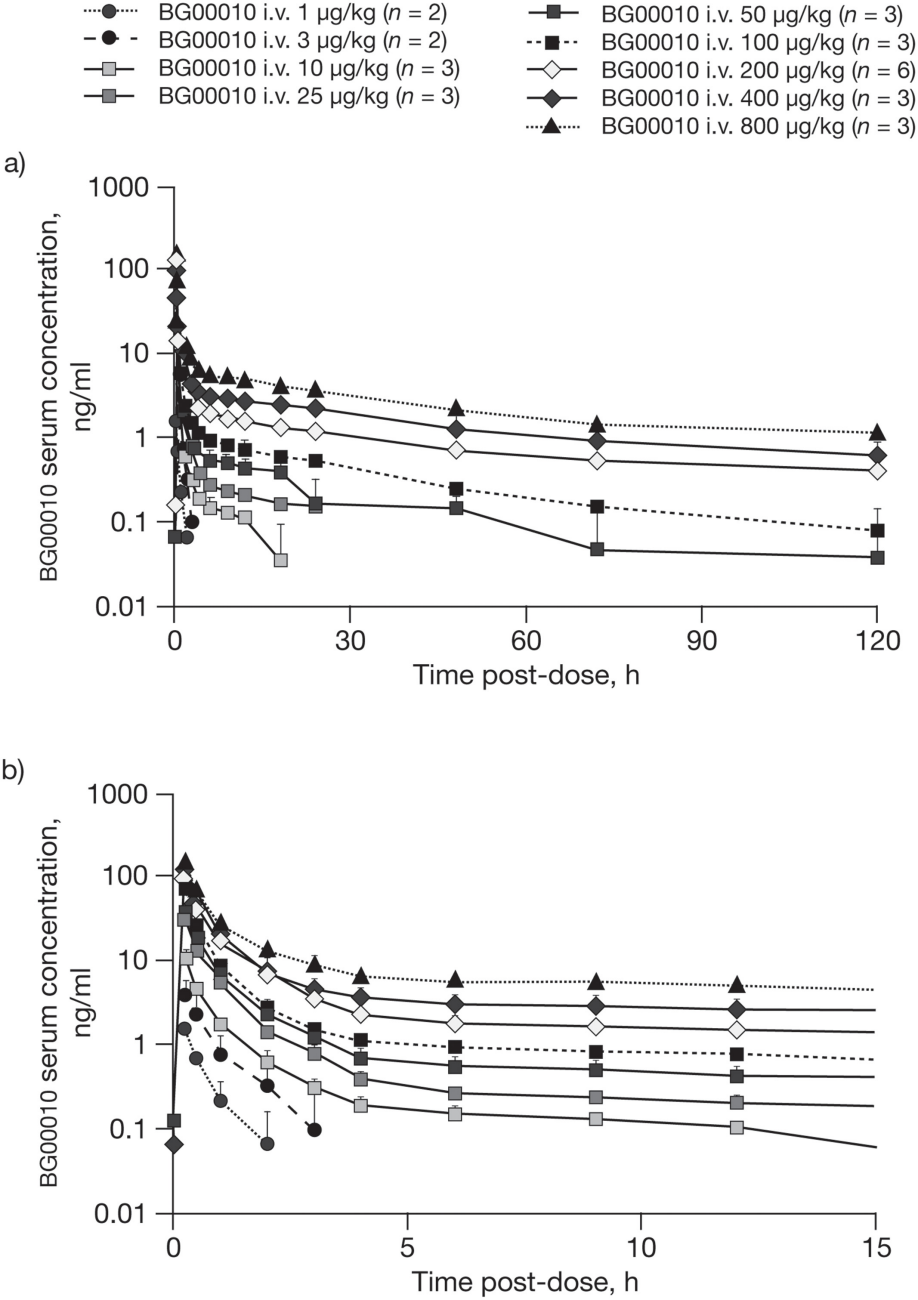
BG00010 serum concentrations over time. Mean (standard deviation) BG00010 serum concentrations over (a) 120 h and (b) 15 h (expanded time axis) following i.v. administration of BG00010. Note that data were only available for two subjects treated with BG00010 25 μg/kg at 9, 12, 18 and 48 h. Where data points are not shown, the mean BG00010 serum concentration was equal to 0.00 ng/ml. h, hours; i.v., intravenous.

The serum concentration—time profiles for the i.v. doses of BG00010 indicate dose-dependent increases in serum BG00010 levels, with relatively low variability within dose cohorts (Fig 5). Peak serum concentrations in the i.v. cohorts were observed at 15 min after treatment, after which there were distinct multiphasic reductions in serum levels, particularly at higher doses. In the first 2 h after treatment, peak serum concentrations generally fell by more than 10-fold, before declining more slowly over the following 5 days.

Pharmacokinetic parameters derived from serum levels of i.v. BG00010 are summarized in Table 3. The relationship between i.v. dose and exposure (AUC_inf_) was nearly linear up to a dose of 200 μg/kg. At i.v. doses above 200 μg/kg, exposure continued to increase, but not in proportion to dose. A similar relationship was observed between i.v. dose and C_max_.

**Table 3:** Summary of pharmacokinetic parameters of i.v. BG00010.

	BG00010 i.v.								
**Dose, μg/kg^a^**	**1**	**3**	**10**	**25**	**50**	**100**	**200**	**400**	**800**
No. of subjects	2^b^	2^c^	3	3	3	3	6	3	3
C_max_, ng/ml, mean (SD)	1.6 (0.2)	4.1 (1.7)	10.8 (2.5)	30.5 (5.8)	36.4 (11.9)	72.8 (5.8)	98.1 (31.4)	114.3 (42.5)	149.0 (42.5)
T_max_, h, mean (SD)	0.3 (0.0)	0.4 (0.0)	0.4 (0.0)	0.4 (0.1)	0.4 (0.0)	0.4 (0.0)	0.5 (0.0)	0.5 (0.0)	0.5 (0.0)
AUC_inf_, ng·h/ml, mean (SD)	0.9 (0.4)	3.3 (1.9)	11.5 (1.2)	31.0 (0.5)	47.5 (9.3)	82.6 (7.4)	201.3 (53.2)	284.7 (100.5)	455.0 (57.2)
Cl, l/h/kg, mean (SD)	1.2 (0.5)	1.1 (0.7)	0.9 (0.1)	0.8 (0.0)	1.1 (0.2)	1.2 (0.1)	1.1 (0.3)	1.6 (0.7)	1.8 (0.2)
V_ss_, l/kg, mean (SD)	0.7 (0.0)	0.9 (0.4)	10.9 (6.8)	9.1 (3.2)	23.5 (14.2)	29.8 (7.9)	52.4 (16.6)	75.5 (20.5)	94.8 (9.3)
t_½_, h, mean (SD)	0.4 (0.3)	0.7 (0.2)	18.1 (9.5)	21.1 (5.7)	27.3 (9.8)	32.3 (9.8)	50.6 (7.7)	45.8 (12.4)	45.1 (3.6)

^a^Pharmacokinetic parameters for BG00010 i.v. 0.3 μg/kg were not calculable due to low serum concentrations of BG00010.

^b^One subject in the BG00010 1 μg/kg cohort was excluded due to low serum concentrations of BG00010.

^c^One subject in the BG00010 3 μg/kg cohort was excluded due to a high serum concentration of BG00010 at predose and low subsequent serum concentrations.

AUC_inf_, area under the serum concentration-time curve from time zero to infinity; Cl, total body clearance; C_max_, maximum observed serum concentration; i.v., intravenous; s.c., subcutaneous; SD, standard deviation; T_max_, time to C_max_; t_½_,terminal half-life; V_ss_, steady-state volume of distribution.

At i.v. BG00010 doses below 200 μg/kg, t_½_ increased as a function of dose, whereas at doses of 200 μg/kg and above, t_½_ stabilized at approximately 45–50 h (see Discussion for interpretation). V_ss_ reached 94.8 l/kg at a dose of 800 μg/kg. Both V_ss_ and Cl appeared to be independent of body weight, and there were no clear differences in pharmacokinetic outcomes between male and female subjects.

For the three subjects treated with BG00010 s.c. 50 μg/kg, serum BG00010 levels were relatively low, as indicated by C_max_ values of 0.1, 0.8, and 0.3 ng/ml (mean C_max_ in i.v. 50 μg/kg cohort: 36.4 ng/ml; Table 4). Relatively rapid absorption was observed, as indicated by T_max_ values of 6.0, 3.0, and 0.5 h, respectively (mean T_max_ in i.v. 50 μg/kg cohort: 0.35 h). Exposure (AUC_inf_) was 10.3 and 24.9 ng·h/ml in the two evaluable subjects in the s.c. cohort. Bioavailability results for these two subjects were 21.7% and 52.4%, based on the mean AUC_inf_ value of 47.5 ng·h/ml for the i.v. 50 μg/kg cohort. The t_1/2_ in these subjects (24.6 and 26.7 h) was comparable to the mean t_1/2_ observed in the i.v. 50 μg/kg cohort (27.3 h).

**Table 4:** Summary of pharmacokinetic parameters of s.c. BG00010 (50 μg/ml).

Subject	T_max_, h	C_max_, ng/ml	t_½_, h	AUC_inf_, ng·h/ml	Bioavailability, %
1^a^	6.0	0.1	–	–	–
2	3.0	0.8	26.7	24.9	52.4
3	0.5	0.3	24.6	10.3	21.7

^a^t_½_, AUC_inf_ and bioavailability could not be determined for one subject.

AUC_inf_, area under the serum concentration-time curve from time zero to infinity; Cl, total body clearance; C_max_, maximum observed serum concentration; i.v., intravenous; s.c., subcutaneous; SD, standard deviation; T_max_, time to C_max_; t_½_,terminal half-life; V_ss_, steady-state volume of distribution.

Pharmacodynamic assessments indicated no significant trends in substance P, monocyte chemoattractant protein-1, or norepinephrine levels in response to BG00010.

## Discussion

In this randomized Phase 1 study, single i.v. doses of BG00010 up to 800 μg/kg and single s.c. doses at 50 μg/kg were generally well tolerated by subjects with sciatica. Most AEs were of mild—moderate severity and not considered to be related to study treatment. In addition, no BG00010 neutralizing antibodies were detected and there were no clinically significant findings or trends in terms of clinical laboratory parameters, vital signs, IENFD, or QST. The study also provided initial insights into the pharmacokinetic and pharmacodynamic profiles of BG00010.

BG00010 was most commonly associated with AEs of feeling hot, pruritus, headache, pruritus generalized, and rash. Of particular note, temperature perception AEs, pruritus AEs, and rash AEs appeared in variable combinations, timings, and durations, but were not regarded by the investigator as particularly troublesome in any subjects. Based on these preliminary findings, such AEs are not considered a likely barrier to treatment if BG00010 is found to be effective in reducing pain. However, it remains to be confirmed whether these AEs would continue with repeated administration.

IENFD monitoring and QST are not considered routine in trials of this type, but were specifically included in this study. Any changes identified with these assessments were not associated with functional effects and were not detected following drug washout. Accordingly, in the current study, IENFD monitoring and QST indicated that a single dose of BG00010 was not associated with any trends for negative effects on normal sensory function.

Pharmacokinetic analyses indicated that, following i.v. administration, there is a rapid initial fall in BG00010 serum levels, followed by a slower, extended decline. This may indicate a substantial inter-compartmental distribution of BG00010. It should be noted that BG00010 pharmacokinetic parameters calculated from concentration—time data at the lower doses may be confounded by the initial distribution kinetics and may not accurately represent true steady-state values. In particular, at i.v. doses below 200 μg/kg, t_½_ increased as a function of dose due to the limited range of concentration—time data at the lower dose levels, which tended to underestimate the true t_½_. Consequently, V_ss_ could only be accurately determined at the higher dose levels, reaching 94.8 l/kg at a dose of 800 μg/kg. The relatively high V_ss_ at doses of 200–800 μg/kg may be due to a high affinity of BG00010 for heparin sulfate moieties in the vasculature.

The Cl of BG00010 following i.v. administration was high, ranging from 0.8 to 1.8 l/h/kg. While Cl and V_ss_ appeared to be independent of body weight, and there were no clear differences in pharmacokinetic outcomes between male and female subjects, a detailed statistical analysis of covariates was not feasible given the small population size. BG00010 s.c. administration resulted in relatively rapid absorption but low BG00010 serum concentrations when compared with i.v. administration of the same dose.

Although some improvements in self-reported ratings of pain and changes in pharmacodynamic outcomes were observed in this single-dose study, there was no overall trend for benefit with BG00010. However, it is important to note that the study was not designed to evaluate efficacy as a primary outcome and that sensitivity in detecting the effects of BG00010 on pain may have been dampened by the administration of single doses only, the continuation of concomitant analgesic therapy, and the relatively small sample size at each dose level. Another artifact of the small sample sizes is the variation in baseline demographics and clinical characteristics between treatment groups; any subsequent pivotal efficacy studies would require much larger group sizes, and could employ techniques such as minimization, to address this issue.

This randomized Phase 1 study was the first-in-human study of BG00010, a first-in-class drug in development for the treatment of pain. It is of particular note that the study was performed in a population with sciatica rather than in healthy volunteers. Although the latter have the advantage of a lack of confounding effects from co-medication, they clearly cannot report any change in spontaneous pain, and the use of experimental pain models in such populations has been of limited utility [15]. This study evaluated subjects with unilateral sciatica, as it is both relatively common and has some mechanistic similarity to the key preclinical studies of BG00010 in rodents with constriction of the sciatic nerve [12–14,16,17]. It is important to note that sciatica is not a purely neuropathic disease and, despite typical radicular symptoms, patients do not necessarily demonstrate nerve root compression, indicating that the condition is probably of mixed neuropathic and nociceptive/inflammatory origin [18]. However, given that this was principally a safety, tolerability, and pharmacokinetic study, such heterogeneity of pathology was not considered to be a significant detractor.

In conclusion, when considered in conjunction with the findings of preclinical efficacy studies [13,14], the safety, tolerability, and pharmacokinetic profiles of BG00010 characterized in this study support the continued clinical development of this agent for the treatment of neuropathic pain.

## Supporting Information

S1 Checklist CONSORT checklist. (DOCX)

S1 Protocol Redacted study protocol. (DOC)
