# Supplementary material for: First-In-Human, Double-Blind, Placebo-Controlled, Randomized, Dose-Escalation Study of BG00010, a Glial Cell Line-Derived Neurotrophic Factor Family Member, in Subjects with Unilateral Sciatica
Source: PLoS One. 2015 May 11;10(5):e0125034. doi: 10.1371/journal.pone.0125034 (PMC4427304; doi:10.1371/journal.pone.0125034)
Supplement: S1 Protocol — (DOC) [file pone.0125034.s002.doc]

| 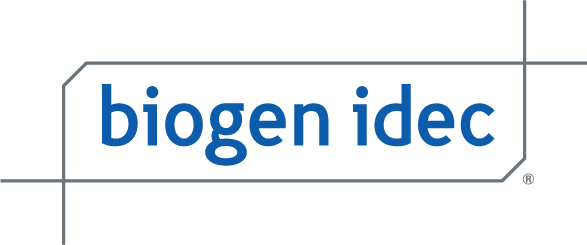 |  |
| --- | --- |
|  | Biogen Idec Inc.  14 Cambridge Center  Cambridge, MA 02142, USA |
| **PROTOCOL NUMBER:** 103NS101 |
|  | Biogen Idec Ltd.  Innovation House  70 Norden Road  Maidenhead Berkshire  SL6 4AY  United Kingdom |
| **STUDY PHASE:** 1 |
|  |  |
| **PROTOCOL TITLE:** A Single-Center, Randomized, Blinded, Placebo-Controlled, Single‑Administration, Sequential‑Cohort, Dose-Escalation Study to Evaluate the Safety, Tolerability, and Pharmacokinetics of BG00010 (Neublastin) Administered to Sciatica Subjects | |
|  |  |
|  |  |
|  |  |
| **DATE:** 27 October 2011  Version 7  FINAL | |
|  |  |

**SIGNATURE PAGE**

<Signatory redacted>

**SIGNATURE PAGE**

<Signatory redacted>

The information contained herein is the confidential and proprietary information of Biogen *Idec, its affiliates, partners and collaborators and may not be used, disclosed or published without the prior written consent of Biogen Idec.*

*Provision of this protocol by Biogen Idec whether in conjunction with the recipient’s review of a publication, manuscript or any other purpose is on a strict confidential basis does not constitute consent for publication on the behalf of Biogen Idec. Separate written consent of Biogen Idec is required prior to any public disclosure by the recipient.*

*Biogen Idec redacts certain sections because the information contained therein is either proprietary or sensitive in nature. Biogen Idec also redacts the names of any individuals including vendors due to privacy issues. The appendices are generally not provided. The protocol includes all the key sections that are relevant to evaluating the study and the manuscript, specifically those sections describing the study objectives and hypotheses, the patient inclusion and exclusion criteria, the study design and procedures, the efficacy and safety measures, and amendments relating to those sections.*

# TABLE OF CONTENTS

[1 Contact List 8](#__RefHeading___Toc307821040)

[2 LIST OF Abbreviations 9](#__RefHeading___Toc307821041)

[3 Synopsis 11](#__RefHeading___Toc307821042)

[4 Study Activities 103NS101: Planned Cohorts A through K 18](#__RefHeading___Toc307821043)

[5 <introduction redacted> 22](#__RefHeading___Toc307821044)

[5.1 Overview of Sciatica 22](#__RefHeading___Toc307821045)

[5.2 Current Therapies for Sciatica 22](#__RefHeading___Toc307821046)

[5.3 <Profile of Previous Experience Redacted> 23](#__RefHeading___Toc307821047)

[5.4 Study Rationale 25](#__RefHeading___Toc307821048)

[5.5 Dose Rationale 25](#__RefHeading___Toc307821049)

[6 Objectives 25](#__RefHeading___Toc307821050)

[6.1 Primary Objective 25](#__RefHeading___Toc307821051)

[7 Study Design 26](#__RefHeading___Toc307821052)

[7.1 Study Overview 26](#__RefHeading___Toc307821053)

[7.1.1 Subcutaneous Administration Substudy 28](#__RefHeading___Toc307821054)

[7.2 Overall Study Duration and Follow-Up 28](#__RefHeading___Toc307821055)

[7.3 Study Stopping Rules 29](#__RefHeading___Toc307821056)

[8 Study Population 30](#__RefHeading___Toc307821057)

[8.1 Inclusion Criteria 30](#__RefHeading___Toc307821058)

[8.2 Exclusion Criteria 30](#__RefHeading___Toc307821059)

[8.3 Screening Log 32](#__RefHeading___Toc307821060)

[9 Enrollment and Randomization Procedures 32](#__RefHeading___Toc307821061)

[9.1 Enrollment Procedures 32](#__RefHeading___Toc307821062)

[9.2 Randomization and Registration Procedures 32](#__RefHeading___Toc307821063)

[9.2.1 Fax Enrollment Form 33](#__RefHeading___Toc307821064)

[9.3 Blinding Procedures 34](#__RefHeading___Toc307821065)

[9.3.1 Unblinding Procedure 34](#__RefHeading___Toc307821066)

[10 STUDY TREATMENT description and allocation 35](#__RefHeading___Toc307821067)

[10.1 <Neublastin description redacted> 35](#__RefHeading___Toc307821068)

[10.2 Placebo 35](#__RefHeading___Toc307821069)

[10.3 Study Treatment Accountability 35](#__RefHeading___Toc307821070)

[11 TREATMENT 36](#__RefHeading___Toc307821071)

[11.1 Treatment Schedule 36](#__RefHeading___Toc307821072)

[11.1.1 Dose Suspension 37](#__RefHeading___Toc307821073)

[11.1.2 Dose Escalation 38](#__RefHeading___Toc307821074)

[11.2 Withdrawal of Subjects From the Study 38](#__RefHeading___Toc307821075)

[11.3 Concomitant Therapy and Procedures 39](#__RefHeading___Toc307821076)

[11.4 Continuation of Treatment 39](#__RefHeading___Toc307821077)

[12 Safety Assessments 39](#__RefHeading___Toc307821078)

[12.1 Clinical Safety Assessments 39](#__RefHeading___Toc307821079)

[12.2 Laboratory Safety Assessments 40](#__RefHeading___Toc307821080)

[12.3 Study Treatment-Specific Safety Assessments 40](#__RefHeading___Toc307821081)

[13 PHARMACokinetic Assessments 41](#__RefHeading___Toc307821082)

[13.1 Pharmacokinetic Assessments 41](#__RefHeading___Toc307821083)

[13.2 Additional Assessments 41](#__RefHeading___Toc307821084)

[14 Schedule of Events 42](#__RefHeading___Toc307821085)

[14.1 Subject Management 42](#__RefHeading___Toc307821086)

[14.2 Tests and Assessments 43](#__RefHeading___Toc307821087)

[14.2.1 Screening Visit (Within 21 Days Prior to the Baseline Visit) 43](#__RefHeading___Toc307821088)

[14.2.2 Baseline Visit (Day -1): Within 72 Hours of Dosing 44](#__RefHeading___Toc307821089)

[14.2.3 Day 0: Predose 45](#__RefHeading___Toc307821090)

[14.2.4 Day 0: Dosing 45](#__RefHeading___Toc307821091)

[14.2.5 Day 0: At 5, 15, 30, and 45 Minutes (±1 Minute) Postdosing 45](#__RefHeading___Toc307821092)

[14.2.6 Day 0: At 1 Hour (±5 Minutes), and at 2, 3, 4, 6, 9, 12 and 18 Hours (±15 Minutes) Postdosing 46](#__RefHeading___Toc307821093)

[14.2.7 Day 1/Hour 24 (±2 Hours) 47](#__RefHeading___Toc307821094)

[14.2.8 Day 2/Hour 48 (±4 Hours) 47](#__RefHeading___Toc307821095)

[14.2.9 Days 3, 5, and 7 (±4 Hours) 48](#__RefHeading___Toc307821096)

[14.2.10 Day 21 (±1 Day) 48](#__RefHeading___Toc307821097)

[14.2.11 Day 28 (‑2 days to +5 days): End of Study/Premature Study Withdrawal Visit 49](#__RefHeading___Toc307821098)

[14.2.12 Day 56 (±4 days): Follow-Up Visit 49](#__RefHeading___Toc307821099)

[15 Safety Definitions, Monitoring, and Reporting 50](#__RefHeading___Toc307821100)

[15.1 Definitions 50](#__RefHeading___Toc307821101)

[15.1.1 Serious Pre-Treatment Event 50](#__RefHeading___Toc307821102)

[15.1.2 Adverse Event 50](#__RefHeading___Toc307821103)

[15.1.3 Serious Adverse Event 50](#__RefHeading___Toc307821104)

[15.2 Monitoring and Recording Events 51](#__RefHeading___Toc307821105)

[15.2.1 Serious Pre-Treatment Events 51](#__RefHeading___Toc307821106)

[15.2.2 Adverse Events 51](#__RefHeading___Toc307821107)

[15.2.3 Serious Adverse Events 51](#__RefHeading___Toc307821108)

[15.2.4 All Events 51](#__RefHeading___Toc307821109)

[15.2.5 Immediate Reporting of Serious Adverse Events 51](#__RefHeading___Toc307821110)

[15.3 Safety Classifications 52](#__RefHeading___Toc307821111)

[15.3.1 Relationship of Events to Study Treatment 52](#__RefHeading___Toc307821112)

[15.3.2 Severity of Events 53](#__RefHeading___Toc307821113)

[15.3.3 Expectedness of Events 53](#__RefHeading___Toc307821114)

[15.4 Prescheduled or Elective Procedures or Routinely Scheduled Treatments 53](#__RefHeading___Toc307821115)

[15.5 Procedures for Handling Special Situations 54](#__RefHeading___Toc307821116)

[15.5.1 Overdose 54](#__RefHeading___Toc307821117)

[15.5.2 Medical Emergency 54](#__RefHeading___Toc307821118)

[15.5.3 Contraception Requirements 54](#__RefHeading___Toc307821119)

[15.5.4 Pregnancy 55](#__RefHeading___Toc307821120)

[15.5.5 Unblinding for Medical Emergencies 55](#__RefHeading___Toc307821121)

[15.5.6 Regulatory Reporting 55](#__RefHeading___Toc307821122)

[15.6 Investigator Responsibilities 56](#__RefHeading___Toc307821123)

[15.7 Biogen Idec Responsibilities 56](#__RefHeading___Toc307821124)

[16 Statistical Statement and Analytical Plan 56](#__RefHeading___Toc307821125)

[16.1 Description of Objectives and Endpoints 56](#__RefHeading___Toc307821126)

[16.1.1 Primary Objective and Endpoints 56](#__RefHeading___Toc307821127)

[16.2 Demography 57](#__RefHeading___Toc307821128)

[16.3 Pharmacokinetics 57](#__RefHeading___Toc307821129)

[16.3.1 Analysis Population 57](#__RefHeading___Toc307821130)

[16.3.2 Methods of Analysis 57](#__RefHeading___Toc307821131)

[16.4 Safety Data 58](#__RefHeading___Toc307821132)

[16.4.1 Analysis Population 58](#__RefHeading___Toc307821133)

[16.4.2 Methods of Analysis 58](#__RefHeading___Toc307821134)

[16.5 Immunogenicity Data 59](#__RefHeading___Toc307821135)

[16.5.1 Analysis Population 59](#__RefHeading___Toc307821136)

[16.5.2 Methods of Analysis 59](#__RefHeading___Toc307821137)

[16.6 Data Analysis for Additional Assessments 59](#__RefHeading___Toc307821138)

[16.7 <Interim Analyses Redacted> 59](#__RefHeading___Toc307821139)

[16.8 Sample Size Considerations 60](#__RefHeading___Toc307821140)

[17 Ethical Requirements 60](#__RefHeading___Toc307821141)

[17.1 Declaration of Helsinki 60](#__RefHeading___Toc307821142)

[17.2 Ethics Committee 60](#__RefHeading___Toc307821143)

[17.3 Subject Information and Consent 61](#__RefHeading___Toc307821144)

[17.4 Subject Data Protection 61](#__RefHeading___Toc307821145)

[18 Administrative Procedures 61](#__RefHeading___Toc307821146)

[18.1 Study Site Initiation 61](#__RefHeading___Toc307821147)

[18.2 Quality Assurance 61](#__RefHeading___Toc307821148)

[18.3 Monitoring of the Study 62](#__RefHeading___Toc307821149)

[18.4 Study Funding 62](#__RefHeading___Toc307821150)

[19 Further Requirements and General Information 62](#__RefHeading___Toc307821151)

[19.1.1 Data Coordinating Center 62](#__RefHeading___Toc307821152)

[19.1.2 Central Laboratories for Laboratory Assessments 62](#__RefHeading___Toc307821153)

[19.2 Drug Safety Review Committee 62](#__RefHeading___Toc307821154)

[19.3 Changes to Final Study Protocol 63](#__RefHeading___Toc307821155)

[19.4 Ethics Committee Notification of Study Completion or Termination 63](#__RefHeading___Toc307821156)

[19.5 Retention of Study Data 63](#__RefHeading___Toc307821157)

[19.6 Study Report Signatory 63](#__RefHeading___Toc307821158)

[20 References 64](#__RefHeading___Toc307821159)

[21 Signed agreement of the study protocol 65](#__RefHeading___Toc307821160)

# LISTING OF FIGURES

[Figure 15-1 Reporting Information for SAEs 52](#__RefHeading___Toc275983418)

# LISTING OF Tables

[Table 9‑1 Study Treatment Randomization Scheme 33](#__RefHeading___Toc275983419)

[Table 11‑1 Planned Cohorts 37](#__RefHeading___Toc275983420)

# Contact List

| Biogen Idec Inc.  14 Cambridge Center  Cambridge, MA 02142  USA |
| --- |

<Personal and confidential information redacted>

# LIST OF Abbreviations

AE adverse event

ALT/SGPT alanine transaminase/serum glutamate pyruvate transaminase

AST/SGOT aspartate transaminase/serum glutamic oxaloacetic transaminase

AUC area under the serum concentration curve

BMI body mass index

°C Degree Celsius (centigrade)

CCI chronic constriction injury

CHO Chinese hamster ovary

CL total body clearance

C_max_ maximum observed serum concentration

CRF Case Report Form

DHA Directions for Handling and Administration

DRG dorsal root ganglion

DSRC Drug Safety Review Committee

ECG electrocardiogram

ECL electrochemiluminescence

ELISA enzyme-linked immunosorbent assay

°F Degree Fahrenheit

GCP Good Clinical Practice

GDNF glial cell line-derived neurotrophic factor

GFRα3 BG00010 receptor

GGT gamma‑glutamyl transferase

HBcAb hepatitis B core antibody

HBsAg hepatitis B surface antigen

HCV hepatitis C virus

HED human equivalent dose

HIV human immunodeficiency virus

ICF informed consent form

ICH International Conference on Harmonisation

IENFD Intra Epidermal Nerve Fiber Density

IV intravenous

LDH lactate dehydrogenase

MCP-1 Monocyte chemoattractant protein-1

MedDRA Medical Dictionary for Regulatory Activities

min minute

NCV nerve conduction velocity

NOAEL No Observed Adverse Effect Level

P3B3 anti-BG00010 antibody

pH acid-base scale

PHI Protected Health Information

PK pharmacokinetic

QST Quantitative Sensory Test

QTc QT interval corrected

RET rearranged during transfection

SABR Safety and Benefit-Risk Management

SAE serious adverse event

SC subcutaneous

SD standard deviation

SF-MPQ Short-Form McGill Pain Questionnaire

SNL spinal nerve ligation

SNRI serotonin noradrenaline reuptake inhibitor

SSRI selective serotonin reuptake inhibitor

SubP Substance P

SUSARs Suspected Unexpected Serious Adverse Reactions

t_½_ terminal half-life

TCA tricyclic antidepressant

TUNEL Terminal deoxynucleotidyl transferase-mediated dUTP Nick End Labeling

VAS Visual Analog Scale

V_ss_ steady state volume of distribution

WBC white blood cell

WOCBP women of child-bearing potential

# Synopsis

| Protocol Number: | 103NS101 |
| --- | --- |
| Version Number: | 7 |
| Protocol Title: | A Single-Center, Randomized, Blinded,  Placebo‑Controlled, Single-Administration, Sequential‑Cohort, Dose-Escalation Study to Evaluate the Safety, Tolerability, and Pharmacokinetics of BG00010 (Neublastin) Administered to Sciatica Subjects |
| Study Phase: | 1 |
| Rationale for the Study: | To evaluate the safety, tolerability, and pharmacokinetics (PK) of BG00010 after single dose intravenous (IV) and subcutaneous (SC) administration to sciatica subjects. BG00010 is a protein that interacts with sensory neurons of the peripheral nervous system to alleviate neuropathic pain. |
| Rationale for Dose and Schedule Selection: | The dose and dosing schedule are based on 2 factors: (a) the 13-week toxicology results, and (b) the projected efficacious human dose.  The 13-week toxicology study performed in non-human primates showed a No Observed Adverse Effect Level (NOAEL) of 4000 μg/kg IV. No adverse events were observed in non-human primates at this dose and it was the highest dose tested. This dose is anticipated to scale to a human equivalent dose (HED) of 1300 μg/kg based on body weight/surface area calculation. The clinical safety margin for the starting human dose of 0.3 μg/kg is 4300-fold.  Allometric scaling was performed with IV doses from rat and monkey data, and exposure estimations from SC efficacy studies performed in rats. A conservative estimate of 10% bioavailability from SC pharmacology studies in rats predicted possible efficacious human dose staring at 37 μg/kg. Therefore, an IV starting dose of 0.3 μg/kg represents approximately 1/100 of the predicted possible efficacious dose.  The selected upper IV dose limit of 800 μg/kg would provide at least a 1.6-fold safety margin based on the NOAEL.  Initially, IV dose cohorts will increase by half-log increments. In IV dose cohorts beyond 10 μg/kg (i.e., at doses approaching the projected efficacious dose) dose escalation will proceed in smaller increments. All subject exposures will be limited to a single dose. Based on the PK of BG00010 in animals, concentration is expected to drop below the bioanalytical quantification limit (0.1 ng/mL) within 6 days.  A single cohort will be administered BG00010 by SC injection to examine a dose level that might provide measurable test article concentrations. A dose of 50 μg/kg represents the highest possible administrable dose due to current formulation limitations. Because of this limitation, the results of this exploration can be used to support, but not reject, the plausibility of SC administration as a viable route of administration. |
| Study Design: | This is a single-center, randomized, blinded, placebo‑controlled, single-administration, sequential‑cohort, dose-escalation study in sciatica subjects. |
| Study Location: | Single study site in Australia |
| Study Objectives: | *Primary*:  To determine the safety/tolerability profile, and systemic PK behavior, and immunogenicity of single IV and SC administrations of BG00010 when administered to sciatica subjects.  The primary endpoints include:   - The number and proportion of subjects with adverse events (AEs). - Assessment of clinical laboratory parameters. - Assessment of vital signs. - Assessment of pain as measured by a Likert numerical pain rating scale. - Longitudinal assessment of the Quantitative Sensory Test (QST; Vibratory, Cool Thermal, and Heat Pain thresholds). - Assessment of Intra Epidermal Nerve Fiber Density (IENFD). - Endpoints for PK assessments. |
| Number of Planned Subjects: | 44 to 84 subjects (4 to 8 subjects per cohort for IV dosing, 4 subjects for the SC administered dose). Each cohort will start off with 4 subjects. Four additional subjects will be added to a cohort if the Drug Safety Review Committee (DSRC) requests that additional subjects be added as the result of a DSRC safety review, or if there is a clinical suspicion of a new sensory abnormality (the determination of sensory function will be made by the DSRC using clinical, neurological, QST, and IENFD data). |
| Study Population: | Subjects with unilateral sciatica who are between 18 and 70 years of age, inclusive; who have a Body Mass Index (BMI) between 18 kg/m^2^ and 32 kg/m^2^, inclusive; and a pain rating of ≥40 mm on the 100 mm Visual Analog Scale (VAS) of the Short-Form McGill Pain Questionnaire (SF‑MPQ). |
| Treatment Groups: | Eleven single-dose cohorts are planned. For each IV cohort, 4 subjects will be randomized to receive 1 IV injection of study treatment (3 subjects will receive BG00010 and 1 subject will receive placebo). For the SC cohort, 4 subjects will be randomized to receive 1 set of SC injections of study treatment (3 subjects will receive BG00010 and 1 subject will receive placebo).  The doses will be escalated according to the following cohort design:   | **Planned Cohorts** | | | | | | --- | --- | --- | --- | --- | | **Cohort** | **BG00010 Dose (μg/kg)** | **Number of Doses of Study Treatment** | **Number of Subjects on BG00010** | **Number of Subjects on Placebo** | | A | 0.3 | 1 | 3 | 1 | | B | 1 | 1 | 3 | 1 | | C | 3 | 1 | 3 | 1 | | D | 10 | 1 | 3 | 1 | | Safety Report submitted to the Ethics Committee (Section 11.1.2) | | | | | | E | 25 | 1 | 3 | 1 | | F | 50 | 1 | 3 | 1 | | Safety Report submitted to the Ethics Committee (Section 11.1.2) | | | | | | G | 100 | 1 | 3 | 1 | | H | 200 | 1 | 3 | 1 | | I | 400 | 1 | 3 | 1 | | J | 800 | 1 | 3 | 1 | | K* | 50 (SC) | 1 | 3 | 1 | | * Recruitment to this cohort will commence anytime after all subjects in Cohort G have completed their Day 56 visit. | | | | |   Only 1 subject will be dosed on any given day. For the second, third, and fourth subjects in each cohort, the Investigator will review data from the preceding subject (including Likert numerical pain rating assessments, physical examination findings, clinical neurological examination findings, AEs/serious adverse events (SAEs), and vital signs) collected during the 24 hours following dosing and determine if the subject’s study treatment is tolerated prior to dosing the next study subject.  The requirement of waiting 24 hours prior to dosing the next subject was based on the animal PK data. As the data suggest that the area under the serum concentration curve (AUC) up to 24 hours accounts for approximately 85% to 99% of the overall AUC following a single‑dose IV or SC administration, a 24‑hour waiting period prior to dosing each subsequent subject is considered sufficient to observe acute treatment-related AEs.  For the main part of the study, 4 additional subjects (3 for BG00010, 1 for placebo) will be added to a cohort if the DSRC requests that additional subjects be added as the result of a DSRC safety review, or if there is a clinical suspicion of a new sensory abnormality (the determination of sensory function will be made by the DSRC using clinical, neurological, QST and, IENFD data).  If a subject experiences a treatment-related increase of 4 points from baseline in one of their pain evaluations, a treatment-related SAE, or a treatment‑related Grade 3 or higher laboratory AE, the Investigator must contact the Biogen Idec Medical Director prior to dosing the next subject. Dosing of a cohort will be suspended until the DSRC completes a safety evaluation (Section 19.2). The decision to continue dosing the cohort will be based on the nature and severity of the SAE or laboratory AE (if applicable), and a review of all available safety data (AEs; all SAEs; vital signs; results for laboratory tests, Likert numerical pain rating assessments, QST, and IENFD). |
| Dose Escalation: | Prior to enrollment into the next IV cohort (the next planned dose level), the DSRC will review unblinded safety data (AEs; all SAEs; vital signs; results for laboratory tests, Likert numerical pain rating assessments, QST, and IENFD) through Day 28, and PK data through Day 5 (or an earlier time point if the concentration drops below the limit of quantification prior to Day 5) from all subjects in the preceding cohorts (see Section 19.2). Enrollment of the next cohort will not begin until the DSRC has approved dosing for that cohort.  Safety data from the SC cohort will not be used as part of the determination for escalation of the IV dose. |
| Visit Schedule: | Subjects will participate in the study for approximately 11 weeks, including a 21-day screening period. Subjects who meet the inclusion/exclusion criteria will enter the inpatient unit on Day -1, the day of the Baseline Visit, within 72 hours prior to administration of study treatment. Subjects will remain in the inpatient unit for at least 48 hours following dosing (Days 0, 1, and 2), and will return to the clinic for postdosing follow‑up visits on Days 3, 5, 7, 21, and 28. Subjects will also have a Follow‑Up Visit at 8 weeks postdosing (Day 56). |
| Safety Assessments: | Physical examinations with vital signs (including systolic and diastolic blood pressure, pulse, and body temperature), clinical neurological examinations, numerical pain rating assessments as measured by an 11-point Likert scale, serial electrocardiograms (ECGs), hematology tests, blood chemistry tests, urinalysis, pregnancy testing, binding antibody analysis, neutralizing antibody analysis, longitudinal QST (Vibratory, Cool Thermal, and Heat Pain thresholds), punch biopsy (IENFD), AE monitoring, and monitoring of concomitant therapy. |
| Pharmacokinetic Assessments: | The PK profile of each dose of study treatment will be constructed by plotting the serum concentration-time curves of BG00010 from blood samples taken prior to and over a 5‑day period following each dose of study treatment. Fifteen serum samples from each subject are to be taken for determination of BG00010 concentration.  The serum concentration of BG00010 will be determined using a chemiluminescent Enzyme-Linked Immunosorbent Assay (ELISA).  The PK parameters anticipated to be calculated and reported include::   - C_max_: maximum observed serum concentration - AUC: area under the serum concentration curve - t_½_: terminal half-life - CL: total body clearance - V_ss_: steady state volume of distribution   If BG00010 concentrations cannot be measured or the measurable time points are not sufficient to calculate all the parameters, only C_max_ and AUC will be reported.  Urine samples will be collected and stored frozen for future analysis. |
| Additional Assessments: | Subjects will complete the VAS of the SF-MPQ. Blood, serum, and plasma will be collected for potential analyses to identify biomarkers that may be indicative of BG00010 activity. Biomarker analysis may include, but not be limited to, Substance P (SubP), monocyte chemoattractant protein-1 (MCP-1), and catecholamines. |
| Statistical Analysis: | Safety and PK parameters will be summarized by cohort using descriptive statistics to provide an initial assessment of the safety and PK properties of BG00010.  The incidence of treatment-emergent AEs and SAEs will be summarized for each dosing cohort overall, by severity, and by relationship to study treatment. SAEs and AEs resulting in withdrawal from the study will be summarized by cohort.  Laboratory results that are outside of the normal range for each parameter will be identified and evaluated for their clinical relevance.  Vital signs will be descriptively examined to determine the incidence of clinically relevant abnormalities, and summarized by cohort.  A listing of subjects with abnormal ECG status will be presented. Changes from baseline will be summarized by cohort.  Change from baseline to endpoint in the severity score from an 11-point Likert scale will be analyzed.  Change from baseline to endpoint in QST will be analyzed. Results will be summarized by cohort and compared with the respective placebo group and a background reference population.  Change from baseline to endpoint in IENFD will be analyzed. Changes will be summarized by cohort and compared with the respective placebo group as a background reference population.  Listings of subjects with normal physical examination and clinical neurological examination status at baseline, but abnormal status at any time after the date of dosing, will be presented.  <Interim analyses plan redacted> |
|  |  |

# Study Activities 103NS101: Planned Cohorts A through K

| **Tests and Assessments** | **Screening**  (within 21 days prior to Day -1) | **Treatment Period** | | | | | | | | | | | | | | | | | |
| --- | --- | --- | --- | --- | --- | --- | --- | --- | --- | --- | --- | --- | --- | --- | --- | --- | --- | --- | --- |
|  |  | **Inpatient** | | | | | | | | | | | | | | | | | |
|  |  | **Baseline**  Day -1 | Day 0 (Dosing Day) | | | | | | | | | | | | | | | | |
|  |  | within  72 hrs  of dosing | Minutes Predose | | | Dose | Minutes Postdosing | | | | Hours Postdosing | | | | | | | | |
|  |  |  |  |  |  |  | (±1 min) | | | | (±5 min) | (±15 min) | | | | | | | |
|  |  |  | -60 | -30 | -15 | 0 | 5 | 15 | 30 | 45 | 1 | 2 | 3 | 4 | 6 | 8 | 9 | 12 | 18 |
| Informed Consent | X |  |  |  |  |  |  |  |  |  |  |  |  |  |  |  |  |  |  |
| Medical History | X |  |  |  |  |  |  |  |  |  |  |  |  |  |  |  |  |  |  |
| Physical Examination | X | X |  |  |  |  |  |  |  |  |  |  |  |  |  |  |  |  |  |
| Vital Signs^1^ | X | X |  |  | X |  | X | X | X | X | X | X | X | X | X |  | X | X | X |
| Body Weight/Height/BMI | X | X^11^ |  |  |  |  |  |  |  |  |  |  |  |  |  |  |  |  |  |
| Clinical Neurological Examination | X | X |  |  |  |  |  |  |  |  |  |  |  |  |  |  |  |  |  |
| VAS of the SF‑MPQ^2^ | X | X |  |  |  |  |  |  |  |  |  |  |  |  |  |  |  |  |  |
| Likert Numerical Pain Rating Assessments^2, 3^ | X^4^ | X^4^ |  | X |  |  |  | X |  | X | X |  |  | X | X |  | X | X |  |
| 12-Lead ECG | X |  | X^15^ |  |  |  | X | X | X |  |  |  |  | X |  |  |  | X |  |
| Hematology | X | X |  |  |  |  |  |  |  |  |  |  |  |  |  |  |  |  |  |
| Blood Chemistry | X^5^ | X |  |  |  |  |  |  |  |  |  |  |  |  |  |  |  |  |  |
| Pregnancy Test | X^6^ | X^12^ |  |  |  |  |  |  |  |  |  |  |  |  |  |  |  |  |  |
| Urinalysis | X | X^13^ |  |  |  |  |  |  |  |  |  |  |  |  |  |  |  |  |  |
| Urine Drug Screen | X | X^13^ |  |  |  |  |  |  |  |  |  |  |  |  |  |  |  |  |  |
| Biomarker Analyses: Blood, Serum, Plasma |  |  | X |  |  |  |  | X^17^ |  |  | X |  |  |  | X |  |  |  |  |
| Hepatitis/HIV | X^7^ |  |  |  |  |  |  |  |  |  |  |  |  |  |  |  |  |  |  |
| Serum BG00010 Concentration (PK) |  |  |  | X |  |  |  | X^19^ | X |  | X | X | X | X | X |  | X | X | X |
| Urine BG00010 Concentration (PK)^18^ |  |  |  |  |  |  |  |  |  |  |  |  |  | X^18^ |  | X^18^ |  | X^18^ |  |
| Serum BG00010 Antibody Assay |  | X |  |  |  |  |  |  |  |  |  |  |  |  |  |  |  |  |  |
| QST | X^8^ | X^8^ |  |  |  |  |  |  |  |  |  |  |  |  |  |  |  |  |  |
| IENFD | X^9^ |  |  |  |  |  |  |  |  |  |  |  |  |  |  |  |  |  |  |
| Randomization |  | X^14^ |  |  |  |  |  |  |  |  |  |  |  |  |  |  |  |  |  |
| Study Treatment Administration |  |  |  |  |  | X^16^ |  |  |  |  |  |  |  |  |  |  |  |  |  |
| AEs/SAEs^3^ and Concomitant Therapy | X^10^ | | | | | Monitor and record throughout the study as per Sections 11.3 and 15. | | | | | | | | | | | | | |

1. Vital signs include systolic and diastolic blood pressure, pulse, and body temperature. The subject must remain in a resting position for 5 minutes prior to having his/her blood pressure taken.
2. VAS (of the SF‑MPQ) and Likert numerical pain rating assessments must be performed prior to blood draws, if applicable.
3. If a subject experiences a treatment-related increase of 4 points from baseline in one of their pain evaluations, a treatment-related SAE, or a treatment-related Grade 3 or higher laboratory AE, the Investigator must contact the Biogen Idec Medical Director prior to dosing the next subject (Section 11.1.1).
4. Seven Likert numerical pain rating sheets are to be given to the subject at the Screening Visit, with instructions to complete one pain assessment per day during the 7 days prior to his/her Baseline Visit. The completed pain assessment sheets are to be collected from the subject at the Baseline Visit.
5. Serum creatinine will be used to estimate creatinine clearance using the Cockroft Gault formula.
6. Serum pregnancy test. Only for women of child-bearing potential (WOCBP).
7. HCV antibodies, HBsAg, HBcAb, and HIV antibodies.
8. QST will be performed twice to minimize subject variance. The second QST should be repeated within 12 hours of the first test. QST at Screening must be performed within 14 days prior to the Baseline Visit. QST will not be performed for Cohort K (50 μg/kg SC).
9. The punch biopsy (IENFD) of the distal unaffected leg will be performed twice to minimize subject variance. The second punch biopsy should be performed on the same leg within 1 hour of the first biopsy. Biopsies will be performed at 10 cm proximal to the malleoli. Screening IENFD maybe performed up to 12 hours prior to dosing. Biopsies will not be performed for Cohort K (50 μg/kg SC).
10. Only serious pre-treatment events and concomitant therapy are to be collected between signing of the ICF and before dosing on Day 0 (Section 15.2.1).
11. Only body weight and BMI will be performed at the Baseline Visit.
12. Urine pregnancy test. Only for women of child-bearing potential (WOCBP).
13. Baseline urinalysis and urine drug screen may be performed on the day of dosing, but results must be available and reviewed by the Investigator prior to dosing.
14. Subjects will be randomized at the Baseline Visit, after all test results have been evaluated and the Investigator has verified that they are eligible per criteria in Sections 8.1 and 8.2.
15. ECG is to be performed 3 times within 15 minutes.
16. The time the IV injection is completed will be considered Hour 0. For the SC cohort, time the last of the series of SC injections is completed will be considered Hour 0.
17. Only serum and plasma will be collected (for potential biomarker analyses) at 15 minutes postdosing.
18. The urine sampling intervals are: 0 - 4 hours; 4 - 8 hours; 8 - 12 hours; and 12 - 24 hours. The TOTAL urine volume excreted during those intervals is to be documented (i.e., complete collections and measurements). The samples will be frozen and stored until required for analysis.
19. The 15 minute post-dose serum sample is for the IV dosing only.

| **Tests and Assessments** | **Follow-Up Period** | | | | | | | |
| --- | --- | --- | --- | --- | --- | --- | --- | --- |
|  | **Inpatient** | | **Outpatient** | | | | | |
|  | Day 1  hr 24  ±2 hrs | Day 2  hr 48  ±4 hrs | Day 3  hr 72  ±4 hrs | Day 5  hr 120  ±4 hrs | Day 7  hr 168  ±4 hrs | Day 21  504 hrs  ±1 day | Day 28^5^  ‑2 days to +5 days | Follow-Up  Day 56^10^ ±4 day |
| Physical Examination | X | X |  |  |  | X | X | X |
| Vital Signs^1^ | X | X | X | X | X | X | X | X |
| Body Weight/Height/BMI |  |  |  |  |  |  | X^6^ |  |
| Clinical Neurological Examination | X | X |  |  |  | X | X | X |
| VAS of the SF‑MPQ^2^ |  |  |  |  |  |  | X | X |
| Likert Numerical Pain Rating Assessments ^2, 3^ | X | X | X | X | X | X | X | X |
| 12-Lead ECG | X | X |  | X |  |  | X |  |
| Hematology | X | X |  | X | X |  | X |  |
| Blood Chemistry | X | X |  | X | X |  | X |  |
| Pregnancy Test |  |  |  |  |  |  | X^7^ |  |
| Urinalysis | X | X |  | X | X |  | X |  |
| Biomarker Analyses: Blood, Serum, Plasma | X |  | X |  |  |  | X^8^ |  |
| Serum BG00010 Concentration (PK) | X | X | X | X |  |  |  |  |
| Urine BG00010 Concentration (PK)^11^ | X^11^ |  |  |  |  |  |  |  |
| Serum BG00010 Antibody Assay |  |  |  |  |  | X | X |  |
| QST | X^4^ |  |  |  |  |  | X^4^ |  |
| IENFD |  |  |  |  |  |  | X^9^ |  |
| AEs/SAEs^3^ and Concomitant Therapy | Monitor and record throughout the study as per Sections 11.3 and 15. | | | | | | | |

Footnotes are provided on the following page.

**Study Activities Table (Follow-Up Period) Footnotes**

1. Vital signs include systolic and diastolic blood pressure, pulse, and body temperature. The subject must remain in a resting position for 5 minutes prior to having his/her blood pressure taken.
2. VAS (of The SF‑MPQ) and Likert numerical pain rating assessments must be performed prior to blood draws, if applicable.
3. If a subject experiences a treatment-related increase of 4 points from baseline in one of their pain evaluations, a treatment-related SAE, or a treatment-related Grade 3 or higher laboratory AE, the Investigator must contact the Biogen Idec Medical Director prior to dosing the next subject (Section 11.1.1).
4. QST will be performed twice to minimize subject variance. The second QST should be repeated within 12 hours of the first test. QST will not be performed for Cohort K (50 μg/kg SC).
5. Day 28 is the End of Study Visit/Premature Study Withdrawal Visit. The tests and assessments specified for Day 28 should also be performed for subjects who withdraw from the study prematurely (within 14 days after withdrawal from the study, if possible). This visit may occur at Day 28 ‑2 days/+5 days for subjects who continue in the study.
6. Only body weight will be performed at the Day 28 visit.
7. Urine pregnancy test. Only for women of child-bearing potential (WOCBP).
8. Collection of blood, serum, and plasma for potential biomarker analyses will only be performed for subjects who withdraw from the study within 48 hours postdosing and complete the tests and assessments for the Premature Study Withdrawal Visit within those 48 hours.
9. The punch biopsy (IENFD) of the distal unaffected leg will be performed twice to minimize subject variance. The second punch biopsy should be performed on the same leg within 1 hour of the first biopsy. Biopsies will be performed at 10 cm proximal to the malleoli. IENFD may be performed on Day 28 ‑2 days/+5 days. Biopsies will not be performed for Cohort K (50 μg/kg SC).
10. The Follow-Up Visit should also be performed for subjects who withdraw from the study prematurely (4 weeks after the Premature Study Withdrawal Visit, if possible). The Follow-Up Visit may occur at Day 56 ±4 days for subjects who continue in the study.
11. The urine sampling intervals are: 0 - 4 hours; 4 - 8 hours; 8 - 12 hours; and 12 - 24 hours. The TOTAL urine volume excreted during those intervals is to be documented (i.e., complete collections and measurements). The samples will be frozen and stored until required for analysis.

# Introduction

BG00010 (also known as neublastin) is a protein in the glial cell line-derived neurotrophic factor (GDNF) family (Baloh et al, 1998).

<Background information redacted>

Biogen Idec proposes the clinical development of BG00010 for the treatment of neuropathic pain.

## Overview of Sciatica

Sciatica is a neuralgic lumbosacral radicular syndrome characterized by radiating leg pain. This pain can involve one or more lower‑limb dermatomes and may be associated with other neurological deficits. Sciatica is relatively common with a lifetime incidence of 13% to 40% in various studies. The annual incidence of sciatica is 1% to 5%, peaking in the fifth decade of life (Frymoyer, 1988; Frymoyer, 1992).

The condition is often debilitating and is a major cause of lost work days, thus creating significant financial burdens for otherwise healthy individuals. About 90% of cases of sciatica are caused by herniated disc with resulting root compression (Koes et al, 2007). The remainder is caused by lateral recess narrowing arising from tumors, or vascular or bony compression.

Sciatica is mainly diagnosed by history and clinical examination, where the patient describes pain radiating along a dermatomal distribution in the leg. The patient may also describe associated sensory symptoms. The physical examination will usually find ipsilateral straight leg raising inducing pain and may detect other neurological deficits localizing to a single root distribution.

## Current Therapies for Sciatica

Most patients with acute sciatica respond to conservative management of their symptoms and the symptoms resolve over weeks to months. Nevertheless, 10% to 40% of patients will develop chronic pain syndrome (Weber et al, 1993). Some require surgical decompression of the compressed nerve root, but a significant proportion will continue to suffer from pain. Pharmacotherapy with tricyclic antidepressants, anticonvulsants, or opiates is commonly used. Epidural steroid injections have also been used for decades, although the data on their efficacy is inconsistent.

## Profile of Previous Experience

<Preclinical Experience with BG00010 redacted>

## Study Rationale

This Phase 1 study is designed to evaluate the safety, tolerability, and pharmacokinetics (PK) of BG00010 after IV administration to sciatica subjects. BG00010 is a protein that interacts with sensory neurons of the peripheral nervous system to alleviate neuropathic pain.

An exploratory substudy of one cohort will evaluate the safety, tolerability, and pharmacokinetics (PK) of BG00010 after subcutanous (SC) administration to sciatica subjects.

## Dose Rationale

The dose and dosing schedule are based on 2 factors: (a) the 13-week toxicology results, and (b) the projected efficacious human dose.

The 13-week toxicology study performed in non-human primates showed a No Observed Adverse Effect Level (NOAEL) of 4000 μg/kg IV. No adverse events were observed in non-human primates at this dose and it was the highest dose tested. This dose is anticipated to scale to a human equivalent dose (HED) of 1300 μg/kg based on body weight/surface area calculation. The clinical safety margin for the starting human dose of 0.3 μg/kg is 4300-fold.

Allometric scaling was performed with IV doses from rat and monkey data, and exposure estimations from SC efficacy studies performed in rats. A conservative estimate of 10% bioavailability from SC pharmacology studies in rats predicted possible efficacious human dose starting at 37 μg/kg. Therefore, a starting dose of 0.3 μg/kg represents approximately 1/100 of a predicted possible efficacious dose.

The selected upper IV dose limit of 800 μg/kg would provide a minimum 1.6-fold safety margin based on the NOAEL.

Initially, IV dose cohorts will increase by half-log increments. In IV dose cohorts beyond 10 μg/kg (i.e., at doses approaching the projected efficacious dose) dose escalation will proceed in smaller increments. All subject exposures will be limited to a single dose. Based on the PK of BG00010 in animals, the concentration is expected to drop below the bioanalytical quantification limit (0.1 ng/mL) within 6 days.

Based on the exposure estimations from SC efficacy studies performed in rats SC, a dose of 50 μg/kg should represent a dose at which BG00010 levels can be detected in the serum and can therefore determine bioavailability relative to IV administration.

# Objectives

## Primary Objective

The primary objective of the study is to determine the safety/tolerability profile, systemic PK behavior, and immunogenicity of single IV and SC administrations of BG00010 to sciatica subjects.

The primary safety/tolerability/immunogenicity endpoints are:

- The number and proportion of subjects with adverse events (AEs).
- Assessment of clinical laboratory parameters.
- Assessment of vital signs.
- Assessment of pain as measured by a Likert numerical pain rating scale.
- Longitudinal assessment of the Quantitative Sensory Test (QST; Vibratory, Cool Thermal, and Heat Pain thresholds).
- Assessment of Intra Epidermal Nerve Fiber Density (IENFD).

The PK parameters anticipated to be calculated and reported include:

- C_max_: maximum observed serum concentration
- AUC: area under the serum concentration curve
- t_½_: terminal half-life
- CL: total body clearance
- V_ss_: steady state volume of distribution

# Study Design

## Study Overview

This is a randomized, blinded, placebo-controlled, single-administration, sequential‑cohort, dose-escalation study of BG00010 in sciatica subjects. This study will be conducted at a single site in Australia. Subjects will participate in the study for approximately 11 weeks, including a 21-day screening period.

Eleven single-dose cohorts (10 IV cohorts of 0.3 μg/kg, 1 μg/kg, 3 μg/kg, 10 μg/kg, 25 μg/kg, 50 μg/kg, 100 μg/kg, 200 μg/kg, 400 μg/kg, and 800 μg/kg, and one SC cohort of 50 μg/kg) are planned. Initially, dose cohorts will increase by half-log increments. In the dose cohorts beyond 10 μg/kg (i.e., at doses approaching the NOAEL) dose escalation will proceed in smaller increments. For each IV cohort, 4 subjects will be randomized to receive 1 IV injection of study treatment (3 subjects will receive BG00010 and 1 subject will receive placebo). For the SC cohort, 4 subjects will be randomized to receive 1 set of SC injections of study treatment (3 subjects will receive BG00010 and 1 subject will receive placebo).

Subjects who meet the inclusion/exclusion criteria will enter the inpatient unit within 72 hours prior to administration of study treatment (Baseline Visit, Day -1). In order to ensure maximal safety of the study subjects, subjects will be monitored in the inpatient unit for at least 48 hours following dosing (Days 0, 1, and 2), and will return to the clinic for postdosing follow-up visits on Days 3, 5, 7, 21, and 28. Subjects will also have a Follow‑Up Visit at 8 weeks postdosing (Day 56). Subject safety will be closely monitored throughout the study period.

Only 1 subject will be dosed on any given day. For the second, third, and fourth subjects in each cohort, the Investigator will review data collected during the 24 hours following dosing (Likert numerical pain rating assessments, physical examination findings, clinical neurological examination findings, AE/SAEs [serious adverse events], and vital signs) from the preceding subject and determine if that subject’s study treatment was tolerated prior to dosing the next study subject.

The requirement of waiting 24 hours prior to dosing the next subject was based on the animal PK data. As the data suggest that the AUC up to 24 hours accounts for approximately 85% to 99% of the overall AUC following a single‑dose IV or SC administration, a 24‑hour waiting period prior to dosing each subsequent subject is considered sufficient to observe acute treatment-related AEs.

If a subject experiences a treatment-related increase of 4 points from baseline in one of their pain evaluations, a treatment-related SAE, or a treatment-related Grade 3 or higher laboratory AE, the Investigator must contact the Biogen Idec Medical Director prior to dosing the next subject. Dosing will be suspended until the Drug Safety Review Committee (DSRC) completes a safety evaluation (Section 19.2). The decision to continue dosing the cohort will be based on the nature and severity of the SAE or laboratory AE (if applicable), and a review of all available safety data (AEs; all SAEs; vital signs; results for laboratory tests, Likert numerical pain rating assessments, QST, and IENFD).

Four additional subjects (3 for BG00010, 1 for placebo) will be added to an IV cohort if the DSRC requests that additional subjects be added as the result of a DSRC safety review, or if there is a clinical suspicion of a new sensory abnormality (the determination of sensory function will be made by the DSRC using clinical, neurological, QST, and IENFD data).

All study site personnel will be blinded to the subject treatment assignments, with the exception of the Pharmacist or designee who is responsible for preparing the study treatment. If an ad hoc DSRC review is required during the course of dosing a cohort, the Investigator may be unblinded to treatment assignment. The Investigator will be unblinded to treatment assignment after Day 28 for each cohort (see Section 9.3).

Prior to enrollment into the next cohort (the next planned dose level), the DSRC will review unblinded safety data (AEs; all SAEs; vital signs; results for laboratory tests, Likert numerical pain rating assessments, QST, and IENFD) through Day 28, and PK data through Day 5 (or an earlier time point if the concentration drops below the limit of quantification prior to Day 5) from all subjects in the preceding cohorts (see Section 19.2). Enrollment of the next cohort will not begin until the DSRC has approved dosing for that cohort.

If exposure for Cohorts A, B, or C exceeds the NOAEL, an ad hoc safety analysis will be performed, and a safety report generated from data collected for all treated Cohorts will be filed with the Ethics Committee prior to initiating dosing for the subsequent cohort.

Two interim safety analyses are planned.

If exposure for Cohorts A, B, or C does not exceed the NOAEL, an interim safety analysis will be performed after all subjects in Cohort D have completed their Day 28 evaluations. A safety report generated from the data collected through Day 28 for Cohorts A through D will be filed with the Ethics Committee prior to initiating dosing of Cohort E. A second interim safety analysis will be completed after all subjects in Cohort F have completed their Day 28 evaluations. A safety report generated from the data collected through Day 28 for Cohorts A through F will be filed with the Ethics Committee prior to initiating dosing of Cohort G.

### Subcutaneous Administration Substudy

An exploratory substudy of one cohort (Cohort K) of 4 subjects (3 BG00010:1 placebo) will evaluate the safety, tolerability, and PK of BG00010 after a single SC administration of 50 μg/kg to sciatica subjects. Recruitment to this cohort will commence anytime after all subjects in the 100 μg /kg IV cohort have completed their Day 56 visit. Data from the SC cohort will not be used as part of the dose escalation determination for the IV dose. QST and IENFD will not be assessed in this cohort. Biopsies will not be performed for IENFD.

## Overall Study Duration and Follow-Up

The study period will consist of Screening, an inpatient treatment period (including Baseline predose testing), and a follow-up period (including both inpatient and outpatient evaluations and an outpatient follow-up period). The overall duration of participation for each subject in the study will be approximately 11 weeks.

##### Screening

Subject eligibility for the study will be determined within 21 days prior to the Baseline Visit. Eligible subjects are to complete Likert numerical pain rating assessments for 7 days prior to their baseline evaluation.

##### Treatment Period

Eligible subjects will report to the study site for admission to the inpatient unit within 72 hours prior to scheduled dosing for a baseline evaluation. An electrocardiogram (ECG) will be performed in triplicate. A Likert numerical pain rating assessment, blood/serum/plasma sampling for potential biomarker analyses, and blood sampling for PK analysis will be performed within 30 minutes prior to scheduled dosing. Vital signs will be performed within 15 minutes prior to scheduled dosing. If the subject remains eligible for study participation, a single dose of study treatment (BG00010 or placebo) will be administered as assigned on Day 0.

##### Follow-Up Period

Subjects will remain in the inpatient unit for at least 48 hours following dosing. During this period, physical examinations; vital signs; clinical neurological examinations; Likert numerical pain rating assessments; serial ECGs; urinalysis; and blood/serum/plasma sampling for hematology, blood chemistry, potential biomarker, and PK analyses will be performed at specified intervals.

Following discharge from the inpatient unit, subjects will be evaluated in the outpatient setting at specified intervals, from Day 3 (72 hours postdosing) through Day 28 (672 hours postdosing). During this period, physical examinations, vital signs, body weight, clinical neurological examinations, Likert numerical pain rating assessments, urinalyses, QST, IENFD, and blood/serum/plasma sampling for hematology, blood chemistry, potential biomarker, antibody, and PK analyses will be performed at specified intervals.

Subjects will also have a Follow-Up Visit at 8 weeks postdosing (Day 56) that will include physical and clinical neurological examinations, Likert numerical pain rating assessments, and visual analog scale (VAS) of the Short-Form McGill Pain Questionnaire (SF MPQ).

##### End of Study

End of study is defined as database lock for the final analysis.

## Study Stopping Rules

Biogen Idec Inc. (Biogen Idec) may terminate this study, after informing the Investigator, at any time. Biogen Idec will notify the Investigator if the study is placed on hold, completed, or closed.

##### Study Stopping Rules

All dosing will cease and the Biogen Idec Medical Director will be contacted immediately if any of the following events occur:

- 2 or more treatment-related SAEs occur in any cohort,
- 2 or more treatment-related Grade 3 or higher (Common Terminology Criteria for Adverse Events, Version 5.0, 2006) laboratory AEs occur in any cohort, or
- A treatment-related significant worsening of sensory function in 2 subjects in any cohort, as determined by the DSRC. A significant change in sensory function will be determined by 2 of any of the following criteria in an individual subject:
- clear worsening of clinical sensory examination (vibratory sensation)
- change in QST of ≥2 standard deviations (of laboratory normative data) from the baseline measurement
- reduction in Intra Epidermal Nerve Fiber Density (IENFD) of ≥2 standard deviations (of laboratory normative data) from the baseline measurement.

# Study Population

## Inclusion Criteria

To be eligible to participate in this study, candidates must meet the following eligibility criteria at the time of enrollment:

1. Must give written informed consent and any authorizations required by local law (e.g., Protected Health Information [PHI]).
2. Must be aged 18 to 70 years old, inclusive, at the time of informed consent.
3. Must have a diagnosis of unilateral sciatica, determined by the Investigator. Sciatica symptoms must be present for 6 or more weeks prior to the Screening Visit.
4. Must have a body mass index (BMI) between 18 kg/m^2^ and 32 kg/m^2^, inclusive.
5. Must rate their pain at ≥40 mm on the 100 mm VAS of the SF-MPQ at the Screening and Baseline Visits.
6. All male subjects and female subjects of child-bearing potential must practice effective contraception during the study and be willing and able to continue contraception for 2 months after their last dose of study treatment. For further details of contraceptive requirements for this study, please refer to Section 15.5.3.

## Exclusion Criteria

Candidates will be excluded from study entry if any of the following exclusion criteria exist at the time of enrollment:

##### Medical History

1. History of malignancy or clinically significant (as determined by the Investigator) allergies, cardiac, endocrinologic, hematologic, hepatic, immunologic, metabolic, urologic, pulmonary, neurologic (not related to sciatica), dermatologic, rheumatic/joint, psychiatric, renal, and/or other major disease.
2. History of signs or symptoms of peripheral neuropathy, other than symptoms of sciatica.
3. History of severe allergic or anaphylactic drug-related reactions.
4. Major surgery within the previous 3 months prior to the Screening Visit.
5. Active pain condition with intensity similar to or worse than that of sciatica.
6. Current generalized myalgia.
7. Fever (body temperature >38°C) or symptomatic viral or bacterial infection within 2 weeks prior to the Baseline Visit.
8. Have a laboratory value at the Screening or Baseline Visits that is outside the normal range, unless it is judged by the Investigator as not clinically significant after appropriate evaluation.
9. Positive for antibody to hepatitis C virus (HCV), positive for hepatitis B surface antigen (HBsAg) or hepatitis B core antibody (HBcAb), or positive for human immunodeficiency virus (HIV) antibody at the Screening Visit.
10. Clinically significant abnormal ECG (12-lead) at the Screening or Baseline Visits, as determined by the Investigator.
11. Have a creatinine clearance <60 mL/min (gender-adjusted, estimated from age and serum creatinine using the Cockroft Gault formula) at the Screening Visit.
12. Subjects who have a marked prolongation of the QT corrected (QTc) interval (i.e., repeated demonstration of a QTc interval >450 msec for females or >430 msec for males) at the Screening or Baseline Visits will not be allowed to enroll into the study.

##### Treatment History

1. Previous participation in a study with neurotrophic factors.
2. Participation in a study with another investigational drug or approved therapy for investigational use within the 3 months prior to the Baseline Visit.
3. Any immunization/vaccination within 1 month prior to the Baseline Visit.
4. Treatment with any prescription medication and/or over-the-counter products such as vitamins or mineral supplements, unless the dose has been stabilized prior to the Baseline Visit. Selective serotonin reuptake inhibitor (SSRI), serotonin noradrenaline reuptake inhibitor (SNRI), and tricyclic antidepressant (TCA) doses must be stable for 4 weeks prior to the Baseline Visit. Gabapentin and pregabalin doses must be stable for 1 week prior to the Baseline Visit.

##### Miscellaneous

1. Female subjects who are pregnant or currently breastfeeding, or who have a positive pregnancy test result at the Screening or Baseline Visits.
2. Significant history of illicit drug or alcohol use or abuse (as determined by the Investigator) within 1 year of the Screening Visit. Subjects who have a positive urine drug test at the Screening or Baseline Visits may be enrolled at the discretion of the Investigator.
3. Blood donation (1 unit or more) within 1 month prior to the Screening Visit.
4. Smoke >5 cigarettes per day.
5. Current enrollment in any other study.
6. Any alcohol use within 24 hours prior to dosing on Day 0.
7. Vigorous exercise (i.e., greater than 30 minutes of aerobic exercise) within 48 hours prior to dosing on Day 0.
8. Unwillingness or inability to comply with the requirements of this protocol, including the presence of any condition (physical, mental, or social) that is likely to affect the subject's returning for follow-up visits on schedule.
9. Other unspecified reasons that, in the opinion of the Investigator or Biogen Idec, make the subject unsuitable for enrollment.

## Screening Log

Participating study sites are required to document all screened candidates initially considered for inclusion in this study. If a subject is excluded from the study, the reasons for exclusion will be documented in the subject’s source documents and on the screening log.

# Enrollment and Randomization Procedures

## Enrollment Procedures

Subjects will be enrolled into the trial at the Baseline Visit, after all baseline assessments have been completed and all relevant laboratory results have been received and reviewed. When it is determined that the subject meets all of the eligibility criteria in Sections 8.1 and 8.2, the subject may be enrolled and a subject identification number may be assigned. These identification numbers should be assigned in numerical order.

Subject identification numbers consist of 6 digits. The first 3 digits are the site number (301), and the second three digits are the subject number (001, 002, etc.). The sample size for this study is 44 to 84 subjects (4 to 8 subjects per cohort). Each cohort will begin with 4 subjects. Four additional subjects will be added to an IV cohort if the DSRC requests that additional subjects be added. Subjects should be assigned randomization numbers in the order that they are enrolled.

## Randomization and Registration Procedures

Subjects will be randomized after the Investigator has verified that they are eligible per criteria in Sections 8.1 and 8.2. Within each cohort, subjects will be randomized to receive BG00010 or placebo in a 3:1 ratio. The computer-generated Master Randomization List will be prepared by Biogen Idec prior to the start of the study and given to the unblinded Pharmacist. Subjects withdrawn from the study may not be replaced.

The Pharmacist will be responsible for storing the Master Randomization List. Only the unblinded Pharmacist and Pharmacy Site Monitor are to have access to the Master Randomization List. The Master Randomization List must be stored in a locked and secured area.

**No subject may begin treatment prior to enrollment and assignment of a unique subject identification number and randomization that is completed by the unblinded Pharmacist.** The subject identification number will designate study treatment and dose according to the predefined randomization schedule. Any subject identification numbers that are assigned will not be reused even if the subject does not receive treatment. Subjects will be enrolled cohort‑by‑cohort. Biogen Idec or its designee will notify the study site in writing when Cohort B and subsequent cohorts are open for enrollment.

Study treatment will be administered according to the following randomization scheme:

Table 9‑1 Study Treatment Randomization Scheme

| **Cohort** | **BG00010 Dose**  **(μg/kg)** | **Number of Doses of Study Treatment** | **Number of Subjects on BG00010** | **Number of Subjects on Placebo** |
| --- | --- | --- | --- | --- |
| A | 0.3 | 1 | 3 | 1 |
| B | 1 | 1 | 3 | 1 |
| C | 3 | 1 | 3 | 1 |
| D | 10 | 1 | 3 | 1 |
| E | 25 | 1 | 3 | 1 |
| F | 50 | 1 | 3 | 1 |
| G | 100 | 1 | 3 | 1 |
| H | 200 | 1 | 3 | 1 |
| I | 400 | 1 | 3 | 1 |
| J | 800 | 1 | 3 | 1 |
| K | 50 (SC) | 1 | 3 | 1 |

### Fax Enrollment Form

As confirmation, the Investigator will provide Biogen Idec with written verification of the subject’s eligibility by fax. The maximum allowable time between randomization and the administration of study treatment is 72 hours.

The site study coordinator should fill out the first section of the Fax Enrollment Form, which will include the subject’s study number, initials, and current weight. The Investigator or Sub-Investigator must provide their signature and date as confirmation that the information entered onto the Fax Enrollment Form is correct.

The Fax Enrollment Form will then be provided to the unblinded Pharmacist, who will use the Master Randomization List to identify the study treatment (BG00010 or Placebo) to which the subject will be randomized. The subject identification number will designate study treatment according to the predefined randomization schedule prepared by Biogen Idec. The Master Randomization List will be provided to the Pharmacist in a special sealed envelope. The Pharmacist should contact the unblinded CRA if the Master Randomization List arrives with the seal broken. A new Master Randomization List will need to be generated.

The unblinded Pharmacist should fill out the second section of the Fax Enrollment Form which will include the subject’s study number, dosage, dose based on weight, and infusion concentration. Once completed, the unblinded Pharmacist should fax this form to the Clinical Trial Manager at Biogen Idec at +1-617-679-3518 and retain a copy of the fax enrollment form in the pharmacy binder.

## Blinding Procedures

This is a randomized, blinded, placebo‑controlled study.

The Pharmacy and the Pharmacy Site Monitor will be unblinded to the assignment of treatment for each subject. In order to maintain the blinding of subject treatment assignments (BG00010 or placebo) all other study site personnel will be blinded to treatment assignment. Physicians, nurses, subjects, and any study site personnel performing subject assessments are NOT to be informed of the subject’s treatment assignment (see Study Reference Guide for blinding guidelines) except in the event of a medical emergency (Section 15.5.2). There will be one Site Monitor (blinded) and one Pharmacy Site Monitor (unblinded) assigned to this study.

However, the Investigator may be unblinded to treatment assignment if an ad hoc DSRC review is required during the course of dosing a cohort due to a subject experiencing a treatment-related increase of 4 points from baseline in one of their pain evaluations, a treatment-related SAE, or a treatment-related Grade 3 or higher laboratory AE.

The Investigator will also be unblinded to treatment assignment after Day 28 for each cohort, so that he/she can participate in the DSRC review of unblinded data for each cohort.

Subjects will be blinded to study treatment assignment throughout the study.

To maintain the study blind, it is imperative that subject treatment assignments are not shared with the subjects, their families, or any member of the study team at the study site, except the unblinded Pharmacist or designee and the Investigator (as noted above).

### Unblinding Procedure

In this study, the Pharmacist or his/her unblinded designee will hold the subjects treatment assignment information. For medical emergencies, the Investigator will be provided with a set of numbered and sealed code-break envelopes that will contain the treatment assignment for each enrolled subject. All treatment assignment information, including code-break envelopes, must be kept in a secure location.

In a medical emergency when knowledge of the subject’s treatment assignment may affect the subject’s clinical care, the Investigator or designee may open the code-break envelope that corresponds to the subject experiencing the event (Section 15.5.5). However, prior to opening the envelope (unblinding), the Investigator or designee should attempt to contact the Biogen Idec Medical Director at Biogen Idec (see study contact information). The date and the reasons for opening the code-break envelope must be submitted to Biogen Idec within 24 hours of the unblinding. Any opened envelopes must be returned to the secure location. Code-break envelopes will be checked at monitoring visits and will be collected at the end of the study.

The Investigator must document the reasons for unblinding in the subject’s source documents. *The Investigator is strongly advised not to divulge the subject’s treatment assignment to any individual not directly involved in managing the medical emergency*

# STUDY TREATMENT description and allocation

Study treatment must be stored in a secure location. Accountability for study treatment is the responsibility of the Investigator. More details concerning this responsibility are included in Section 10.3.

Study treatment must only be dispensed by a Pharmacist or medically qualified staff. Study treatment is to be dispensed only to subjects enrolled in this study. Once study treatment is prepared for a subject, it can only be administered to that subject. Study treatment vials are for one‑time use only; any study treatment remaining in the vial should not be used for another subject.

Study site staff should refer to the Directions for Handling and Administration (DHA) located in the Study Reference Manual for specific instructions on the handling and administration of the study treatment. The DHA supersedes all other references (e.g., Investigator’s Brochure).

## <BG00010 (Neublastin) descripton redacted>

## Placebo

Saline provided by the study site will be used as placebo.

## Study Treatment Accountability

The study site must maintain accurate records demonstrating dates and amount of study treatment received, to whom dispensed (subject‑by‑subject accounting), and accounts of any study treatment accidentally or deliberately destroyed.

Unless otherwise notified, all used and unused vials must be saved for study treatment accountability. At the end of the study, reconciliation must be made between the amount of study treatment supplied, dispensed, and subsequently destroyed. A written explanation will be provided for any discrepancies. After reconciliation, the Investigator must destroy all unused vials of study treatment as instructed by Biogen Idec.

If any study treatment supplies are to be destroyed at the site, the institution/Principal Investigator must obtain prior approval by Biogen Idec. After such destruction, the institution/Principal Investigator must notify Biogen Idec, in writing, of the method of destruction, the date of destruction, and the location of destruction.

# TREATMENT

## Treatment Schedule

Eleven single-dose cohorts are planned (10 IV and 1 SC). For each cohort, 4 subjects will be randomized to receive 1 adminstration of study treatment (3 subjects will receive BG00010 and 1 subject will receive placebo).

For the dose escalation part of the study, study treatment will be administered as an IV injection by the Investigator or designee.

For the SC administration substudy, study treatment will be administered as up to 4 SC injections (depending on body weight).

BG00010 doses will be escalated according to the cohort design presented in Table 11-1.

Table 11‑2 Planned Cohorts

| **Cohort** | **BG00010 Dose**  **(μg/kg)** | **Number of Doses of Study Treatment** | **Number of Subjects on BG00010** | **Number of Subjects on Placebo** |
| --- | --- | --- | --- | --- |
| A | 0.3 | 1 | 3 | 1 |
| B | 1 | 1 | 3 | 1 |
| C | 3 | 1 | 3 | 1 |
| D | 10 | 1 | 3 | 1 |
| Safety Report submitted to the Ethics Committee (Section 11.1.2) | | | | |
| E | 25 | 1 | 3 | 1 |
| F | 50 | 1 | 3 | 1 |
| Safety Report submitted to the Ethics Committee (Section 11.1.2) | | | | |
| G | 100 | 1 | 3 | 1 |
| H | 200 | 1 | 3 | 1 |
| I | 400 | 1 | 3 | 1 |
| J | 800 | 1 | 3 | 1 |
| K* | 50 (SC) | 1 | 3 | 1 |
| * Recruitment to this cohort will commence anytime after all subjects in Cohort G (100 μg/kg) have completed their Day 56 visit. | | | | |

Four additional subjects will be added to an IV cohort if the DSRC requests that additional subjects be added as the result of a DSRC safety review, or if there is a clinical suspicion of a new sensory abnormality (the determination of sensory function will be made by the DSRC using clinical, neurological, QST, and IENFD data).

### Dose Suspension

The Investigator must contact the Biogen Idec Medical Director prior to dosing the next subject if a subject experiences a treatment-related increase of 4 points from baseline in one of their pain evaluations, a treatment-related SAE, or a treatment-related Grade 3 or higher laboratory AE.

Dosing of a cohort will be suspended until the DSRC completes a safety evaluation (Section 19.2). The decision to continue dosing the cohort will be based on the nature and severity of the SAE or laboratory AE (if applicable), and a review of all available safety data (AEs; all SAEs; vital signs; results for laboratory tests, Likert numerical pain rating assessments, QST, and IENFD).

Refer to Section 7.3 for Study Stopping Rules.

### Dose Escalation

Prior to enrollment into the next IV cohort (the next planned dose level), the DSRC will review unblinded safety data (AEs; all SAEs; vital signs; results for laboratory tests, Likert numerical pain rating assessments, QST, and IENFD) through Day 28, and PK data through Day 5 (or an earlier time point if the concentration drops below the limit of quantitation prior to Day 5) from all subjects in the preceding cohorts (see Section 19.2). Enrollment of the next cohort will not begin until the DSRC has approved dosing for that cohort.

If serum exposure for Cohorts A, B, or C exceeds the NOAEL, an ad hoc safety analysis will be performed, and a safety report generated from data collected for all treated Cohorts will be filed with the Ethics Committee prior to initiating dosing for the subsequent cohort.

Two interim safety analyses are planned. If serum exposure for Cohorts A, B, or C does not exceed the NOAEL, an interim safety analysis will be performed after all subjects in Cohort D have completed their Day 28 evaluations. A safety report generated from the data collected through Day 28 for Cohorts A through D will be filed with the Ethics Committee prior to initiating dosing of Cohort E. A second interim safety analysis will be completed after all subjects in Cohort F have completed their Day 28 evaluations. A safety report generated from the data collected through Day 28 for Cohorts A through F will be filed with the Ethics Committee prior to initiating dosing of Cohort G.

Safety data from the SC cohort will not be used as part of the determination for escalation of the IV dose.

## Withdrawal of Subjects From the Study

Subjects must be withdrawn from the study for any one of the following reasons:

- The subject desires to discontinue participation in this study.
- The subject is unwilling or unable to comply with the protocol.
- Other unspecified reasons that, in the opinion of the Investigator, make the subject unsuitable to continue study participation.

Subjects who prematurely withdraw from the study postdosing should be encouraged to complete the tests and evaluations for the Day 28 visit within 14 days after withdrawal from the study, as well as the Follow-Up Visit 28 days later. If the subject withdraws within 48 hours postdosing and completes the tests/assessments for the Premature Study Withdrawal Visit within those 48 hours, blood, serum, and plasma for potential biomarker analyses will also be collected. Subjects who prematurely withdraw postdosing may not be replaced. However, subjects who prematurely withdraw prior to dosing may be replaced. The reasons for the subject’s withdrawal from the study must be recorded in the subject’s Case Report Form (CRF).

## Concomitant Therapy and Procedures

A concomitant therapy is any drug or substance, including nonprescription drugs, herbal preparations, and vitamins, administered from the Screening Visit until the Follow-Up Visit (Day 56).

Concomitant treatment with narcotic and non-narcotic analgesics, acetaminophen (not to exceed 4 g/day), non-steroidal anti-inflammatory drugs, aspirin, and over-the-counter products such as vitamins or mineral supplements is allowed if the dosing of these medications/supplements has been stabilized prior to the Baseline Visit and remains stable throughout the study. Concomitant treatment with previously prescribed TCAs, SSRIs, and SNRIs is allowed if the dosing of these medications has been stable for 4 weeks prior to the Baseline Visit and remains stable throughout the study. Concomitant treatment with gabapentin and pregabalin is allowed if the dosing of these medications has been stable for 1 week prior to the Baseline Visit and remains stable throughout the study.

Subjects should be instructed not to start taking any new medications, including nonprescription drugs and herbal preparations, unless they have received permission from the Investigator.

A concomitant procedure is any therapeutic intervention (e.g., surgery/biopsy, physical therapy) or diagnostic assessment (e.g., blood gas measurement, bacterial cultures) performed from the Screening Visit until the Follow-Up Visit, unless the subject is being followed for study‑related toxicity.

The use of concomitant therapies or procedures defined above must be recorded on the subject’s CRF, according to instructions for CRF completion. AEs related to administration of these therapies or procedures must be documented on the appropriate CRF.

## Continuation of Treatment

There is no provision for additional courses of BG00010 provided by Biogen Idec beyond the treatment defined in this protocol.

# Safety Assessments

## Clinical Safety Assessments

The following clinical assessments will be performed to assess the safety profile of BG00010:

- complete physical examinations
- measurement of vital signs (including systolic and diastolic blood pressure, pulse, and body temperature)
- clinical neurological examinations including a motor examination, a deep tendon reflex examination, and a sensory examination (joint position sensation, vibration sensation, pinprick sensation, and temperature sensation)
- numerical pain rating assessments as measured by an 11-point Likert scale (Likert, 1952)
- ECG (12-lead)
- longitudinal assessment of QST (Vibratory, Cool Thermal, and Heat Pain thresholds)
- punch biopsy (IENFD)
- monitoring of AEs, SAEs, and concomitant therapy.

## Laboratory Safety Assessments

The following laboratory tests will be performed at a local laboratory to assess the safety profile of BG00010:

- hematology: hemoglobin, hematocrit, red blood cell count, white blood cell (WBC) count (with differential), and platelet count.
- blood chemistry: sodium, potassium, chloride, bicarbonate, urea, creatinine, glucose, alkaline phosphatase, total bilirubin, alanine transaminase/serum glutamate pyruvate transaminase (ALT/SGPT), aspartate transaminase/serum glutamic oxaloacetic transaminase (AST/SGOT), lactate dehydrogenase (LDH), gamma-glutamyl transferase (GGT), serum lipase.
- urinalysis: color, urobilinogen, specific gravity, pH, protein, bilirubin, glucose, blood, ketones, nitrite, microscopic WBC count, microscopic red blood cell count.
- serum and urine pregnancy tests for all women of child-bearing potential (WOCBP), as defined in Section 15.5.3.

## Study Treatment-Specific Safety Assessments

The following tests will be performed to determine the study treatment-specific safety of BG00010:

- Binding Antibody Assay: The presence of anti-BG00010 antibodies will be determined using a tiered assay approach involving a screening assay and a confirmation assay, followed by titration of positive samples. The presence of anti-BG00010 antibodies in human serum will be determined using an electrochemiluminescent (ECL) assay format. Assay cut points will be determined using appropriate statistical methods. Screening positive samples will be further evaluated by a confirmation assay. Confirmed positive samples will be characterized to determine titer values.
- Neutralizing Antibody Assay: The samples that are identified to be positive for binding antibodies will be further evaluated in the neutralizing antibody assay. The neutralizing antibody assay is based on the biological potency assay that mimics the proposed mode of action of the drug. Specifically, the assay measures the ability of BG00010 to bind to and activate the extracellular GFRα3 receptor, and thus inducing phosphorylation of the intracellular RET receptor tyrosine kinase. In the presence of neutralizing antibodies an inhibition of RET phosphorylation is observed. A sample is designated as positive for neutralizing antibodies if the observed inhibition is above the pre-determined assay cut point.

Antibody samples will be tested at a qualified analytical testing laboratory. A description of the collection and shipping procedures for the samples is provided in the Study Reference Guide.

# PHARMACokinetic Assessments

## Pharmacokinetic Assessments

The PK profile of each dose of study treatment will be assessed by measurement of BG00010 in serum taken over a 5-day period following each dose of study treatment. Fifteen serum samples from each subject are to be collected for determination of BG00010 concentration.

The concentration of BG00010 in serum will be determined using a chemiluminescent Enzyme‑Linked Immunosorbent Assay (ELISA). The ELISA assay is based on the binding of BG00010 to immobilized anti-BG00010 antibody (P3B3). The captured BG00010 is incubated with biotinylated P3B3 antibody and the BG00010-bound biotinylated P3B3 antibody is then detected using streptavidin conjugated to horseradish peroxidase, which upon addition of Luminol substrate, produces a chemiluminescent signal.

The total urine volume excreted during intervals 0 to 4 hours; 4 to 8 hours; 8 to 12 hours; and 12 to 24 hours postdose are to be documented (i.e., complete collections and measurements). The samples will be frozen and stored for future analysis.

PK samples will be tested at a qualified analytical testing laboratory. A description of the collection and shipping procedures for the samples is provided in the Study Reference Guide.

## Additional Assessments

Subjects will complete the VAS of the SF-MPQ at Screening and Baseline to determine eligibility, Day 56, and at the End of Study/Premature Study Withdrawal Visit.

Blood, serum, and plasma samples will be collected for analyses to identify potential biomarkers that may indicate BG00010 activity. Biomarker analysis may include, but not be limited to, Substance P (SubP), monocyte chemoattractant protein-1 (MCP-1), and catecholamines.

Serum and plasma samples (with added protease inhibitors) for protein analysis will be collected 60 minutes predose, and 15 minutes, 1 hour, 6 hours, 24 hours, and 72 hours postdose.

Whole blood (in PAXgene tubes) for potential transcript profiling and plasma for global proteomics will be collected 60 minutes predose, and 1 hour, 6 hours, 24 hours, and 72 hours postdose.

Serum and plasma samples will be analyzed for targeted biomarkers, and whole blood and additional plasma samples will be banked for possible future exploratory analyses.

A description of the collection and shipping procedures for the samples is provided in the Study Reference Guide.

# Schedule of Events

A written, signed Informed Consent Form (ICF) and all authorizations required by local law (e.g., PHI in North America) must be obtained prior to performing any tests or assessments under this protocol.

## Subject Management

Male subjects and female subjects of child-bearing potential must practice effective contraception during the study and continue contraception for 2 months after their last dose of study treatment. If a female subject becomes pregnant, study treatment must be discontinued immediately. For detailed information on contraception requirements and pregnancy, please refer to Sections 15.5.3 and 15.5.4.

Subjects will be instructed to complete one pain assessment per day, using Likert numerical pain rating sheets, during the 7 days prior to his/her Baseline Visit.

Subjects will enter the inpatient unit on Day -1, the day of the Baseline Visit, (within 72 hours prior to administration of study treatment) and will remain in the unit for at least 48 hours following dosing (Days 0, 1, and 2). Six postdosing follow-up visits will occur in the clinic: Days 3, 5, 7, 21, and 28. Subjects will also have a Follow-Up Visit on Day 56.

On the morning of dosing, subjects will be served a standard breakfast. The IV or SC injections will be given between 45 and 90 minutes following this meal. Subjects will then fast for approximately 3 hours after which time a standard lunch will be served. A standard dinner will be served approximately 6 hours after lunch. A standard breakfast will be given the following morning. All subjects will be served identical meals during the 48 hours following dosing.

Beverages will be permitted, in moderation, upon request. Subjects will be allowed 2 caffeinated beverages per day while confined to the unit.

Subjects will be monitored by serial ECG.

Vigorous exercise (i.e., aerobic exercise for greater than 30 minutes) is prohibited from 48 hours prior to administration of study treatment until 24 hours after administration of study treatment. Alcohol use is prohibited from 24 hours prior to administration of study treatment until 1 week after administration of study treatment (Day 7), and for 24 hours prior to other visits. Alcohol use is restricted to moderate consumption (i.e., up to an equivalent of 2 pints of beer in any 24‑hour period) through the end of the study. Subjects who have a positive urine drug test at the Screening or Baseline Visit may be enrolled at the discretion of the Investigator.

Subjects must be instructed NOT to donate blood, plasma, or any other blood products for 3 months following administration of study treatment.

## Tests and Assessments

### Screening Visit (Within 21 Days Prior to the Baseline Visit)

The following tests and assessments will be performed at the Screening Visit within 21 days prior to the Baseline Visit in order to determine subject eligibility and/or baseline values:

- written informed consent, and PHI authorization (if required by local law)
- medical history, including history of drug abuse and current medications
- physical examination
- vital signs (systolic and diastolic blood pressure, pulse, body temperature). The subject must remain in a resting position for 5 minutes prior to having his/her pulse and blood pressure taken.
- body weight, height, and BMI
- clinical neurological examination
- VAS of the SF‑MPQ (must be performed prior to blood draws)
- Likert numerical pain rating assessment (must be performed prior to blood draws). Likert numerical pain rating sheets will be given to the subject with instructions to complete one pain assessment per day during the 7 days prior to his/her Baseline Visit.
- ECG, 12-lead
- collection of serum and whole blood for hematology and blood chemistry tests (serum creatinine will be used to estimate creatinine clearance using the Cockroft Gault formula)
- collection of blood for serum pregnancy test (WOCBP only)
- collection of urine for urinalysis and urine drug screen
- collection of blood for hepatitis/HIV testing (HCV antibodies, HBsAg, HBcAb, and HIV antibodies)
- QST will be performed twice to minimize subject variance. The second QST should be repeated within 12 hours of the first test. (Must be performed within 14 days prior to the Baseline Visit) QST will not be performed in Cohort K.
- punch biopsy (IENFD) of the distal unaffected leg will be performed twice to minimize subject variance. The second punch biopsy should be performed on the same leg within 1 hour of the first biopsy. Biopsies will be performed at 10 cm proximal to the malleoli. NOTE: The IENFD may be performed up to 12 hours prior to dosing. Punch biopsies and IENFD will not be performed in Cohort K.
- monitoring/recording of serious pre-treatment events
- monitoring/recording of concomitant therapy

### Baseline Visit (Day -1): Within 72 Hours of Dosing

Subjects will report to the study site within 72 hours prior to dosing. Subjects will remain in the inpatient unit until completion of the Day 2 (hour 48) postdosing evaluations.

The following tests and assessments are to be performed within 72 hours prior to dosing:

- physical examination
- vital signs (systolic and diastolic blood pressure, pulse, body temperature). The subject must remain in a resting position for 5 minutes prior to having his/her pulse and blood pressure taken.
- body weight and BMI
- clinical neurological examination
- VAS of the SF‑MPQ (must be performed prior to blood draws)
- Likert numerical pain rating assessment (must be performed prior to blood draws). Likert numerical pain assessment sheets, completed by the subject during the 7 days prior to the Baseline Visit, will be collected.
- collection of serum and whole blood for hematology and blood chemistry tests
- collection of urine for urine pregnancy test (WOCBP only)
- collection of urine for urinalysis and urine drug screen. Urine collection may be performed on the day of dosing, but urinalysis results must be reviewed prior to dosing.
- collection of serum for anti-BG00010 antibody assay
- QST will be performed twice to minimize subject variance. The second QST should be repeated within 12 hours of the first test. QST will not be performed in Cohort K.
- randomization
- monitoring/recording of serious pre-treatment events
- monitoring/recording of concomitant therapy

### Day 0: Predose

The following tests and assessments are to be performed within 60 minutes prior to dosing:

- ECG, 12-lead to be performed 3 times within 15 minutes
- collection of whole blood, serum, and plasma for potential biomarker analyses (Section 13.2)
- monitoring/recording of serious pre-treatment events
- monitoring/recording of concomitant therapy

The following tests and assessments are to be performed within 30 minutes prior to dosing:

- Likert numerical pain rating assessment (must be performed prior to blood draws)
- collection of serum for determination of BG00010 concentration (PK)
- monitoring/recording of serious pre-treatment events
- monitoring/recording of concomitant therapy

The following tests and assessments are to be performed within 15 minutes prior to dosing:

- vital signs (systolic and diastolic blood pressure, pulse, body temperature). The subject must remain in a resting position for 5 minutes prior to having his/her pulse and blood pressure taken.
- monitoring/recording of serious pre-treatment events
- monitoring/recording of concomitant therapy

### Day 0: Dosing

- administer study treatment (the time that the IV injection is completed will be considered Hour 0, for the SC cohort, the time the last of the series of SC injections is completed will be considered Hour 0)
- monitoring/recording of AEs/SAEs
- monitoring/recording of concomitant therapy

### Day 0: At 5, 15, 30, and 45 Minutes (±1 Minute) Postdosing

The following tests and assessments must be performed at 5, 15, 30, and 45 minutes (±1 minute) after dosing:

- vital signs (systolic and diastolic blood pressure, pulse, body temperature). The subject must remain in a resting position for 5 minutes prior to having his/her pulse and blood pressure taken.
- Likert numerical pain rating assessment (must be performed prior to blood draws; at 15 and 45 minutes postdosing only)
- ECG, 12-lead (at 5, 15, and 30 minutes postdosing only)
- collection of serum and plasma for potential biomarker analyses (at 15 minutes postdosing only, Section 13.2)
- collection of serum for determination of BG00010 concentration (PK, at 15 [IV dosing only] and 30 minutes postdosing only)
- monitoring/recording of AEs/SAEs
- monitoring/recording of concomitant therapy

### Day 0: At 1 Hour (±5 Minutes), and at 2, 3, 4, 6, 9, 12 and 18 Hours (±15 Minutes) Postdosing

The following tests and assessments must be performed at 1 hour (±5 minutes) after dosing and at 2, 3, 4, 6, 9, 12, and 18 hours (±15 minutes) after dosing:

- vital signs (systolic and diastolic blood pressure, pulse, body temperature). The subject must remain in a resting position for 5 minutes prior to having his/her pulse and blood pressure taken.
- Likert numerical pain rating assessment (must be performed prior to blood draws; at 1, 4, 6, 9, and 12 hours postdosing only)
- ECG, 12-lead (at 4 and 12 hours postdosing only)
- collection of whole blood, serum, and plasma for potential biomarker analyses (at 1 and 6 hours postdosing only, Section 13.2)
- collection of serum for determination of BG00010 concentration (PK)
- collection of urine at 0 to 4 hours, 4 to 8 hours, and 8 to 12 hours (the total urine volume excreted during these intervals is to be documented)
- monitoring/recording of AEs/SAEs
- monitoring/recording of concomitant therapy

### Day 1/Hour 24 (±2 Hours)

The following tests and assessments must be performed at 24 hours (±2 hours) after dosing:

- physical examination
- vital signs (systolic and diastolic blood pressure, pulse, body temperature). The subject must remain in a resting position for 5 minutes prior to having his/her pulse and blood pressure taken.
- clinical neurological examination
- Likert numerical pain rating assessment (must be performed prior to blood draws)
- ECG, 12-lead
- collection of serum and whole blood for hematology and blood chemistry tests
- collection of urine for urinalysis
- collection of whole blood, serum, and plasma for potential biomarker analyses (Section 13.2)
- collection of serum for determination of BG00010 concentration (PK)
- QST will be performed twice to minimize subject variance. The second QST should be repeated within 12 hours of the first test. QST will not be performed in Cohort K.
- collection of urine at 12 to 24 hours (the total urine volume excreted during this interval is to be documented)
- monitoring/recording of AEs/SAEs
- monitoring/recording of concomitant therapy

### Day 2/Hour 48 (±4 Hours)

The following tests and assessments must be performed at 2 days/48 hours (±4 hours) after dosing:

- physical examination
- vital signs (systolic and diastolic blood pressure, pulse, body temperature). The subject must remain in a resting position for 5 minutes prior to having his/her pulse and blood pressure taken.
- clinical neurological examination
- Likert numerical pain rating assessment (must be performed prior to blood draws)
- ECG, 12-lead
- collection of serum and whole blood for hematology and blood chemistry tests
- collection of urine for urinalysis
- collection of serum for determination of BG00010 concentration (PK)
- monitoring/recording of AEs/SAEs
- monitoring/recording of concomitant therapy

### Days 3, 5, and 7 (±4 Hours)

The following tests and assessments must be performed at 3 days/72 hours, 5 days/120 hours, and 7 days/168 hours (±4 hours) after dosing:

- vital signs (systolic and diastolic blood pressure, pulse, body temperature). The subject must remain in a resting position for 5 minutes prior to having his/her pulse and blood pressure taken.
- Likert numerical pain rating assessment (must be performed prior to blood draws)
- ECG, 12-lead (Day 5 only)
- collection of serum and whole blood for hematology and blood chemistry tests (Days 5 and 7 only)
- collection of urine for urinalysis (Days 5 and 7 only)
- collection of whole blood, serum, and plasma for potential biomarker analyses (Section 13.2, Day 3 only)
- collection of serum for determination of BG00010 concentration (PK, Days 3 and 5 only)
- monitoring/recording of AEs/SAEs
- monitoring/recording of concomitant therapy

### Day 21 (±1 Day)

The following tests and assessments must be performed at 21 days/504 hours (±1 day) after dosing:

- physical examination
- vital signs (systolic and diastolic blood pressure, pulse, body temperature). The subject must remain in a resting position for 5 minutes prior to having his/her pulse and blood pressure taken.
- clinical neurological examination
- Likert numerical pain rating assessment (must be performed prior to blood draws)
- collection of serum for anti-BG00010 antibody assay
- monitoring/recording of AEs/SAEs
- monitoring/recording of concomitant therapy

### Day 28 (‑2 days to +5 days): End of Study/Premature Study Withdrawal Visit

The following tests and assessments must be performed at 28 days/‑2 days to +5 days after dosing. Subjects who withdraw from the study prematurely should complete these tests and assessments within 14 days after withdrawal from the study, if possible:

- physical examination
- vital signs (systolic and diastolic blood pressure, pulse, body temperature). The subject must remain in a resting position for 5 minutes prior to having his/her pulse and blood pressure taken.
- body weight
- clinical neurological examination
- VAS of the SF‑MPQ (must be performed prior to blood draws)
- Likert numerical pain rating assessment (must be performed prior to blood draws)
- ECG, 12-lead
- collection of serum and whole blood for hematology and blood chemistry tests
- collection of urine for urine pregnancy test (WOCBP only)
- collection of urine for urinalysis
- collection of whole blood, serum, and plasma for potential biomarker analyses (only for subjects who withdraw from the study within 48 hours postdosing and complete the tests and assessments for the Premature Study Withdrawal Visit within those 48 hours, Section 13.2)
- collection of serum for anti-BG00010 antibody assay
- QST will be performed twice to minimize subject variance. The second QST should be repeated within 12 hours of the first test. QST will not be performed in Cohort K.
- punch biopsy (IENFD) of the distal unaffected leg will be performed twice to minimize subject variance. The second punch biopsy should be performed on the same leg within 1 hour of the first biopsy. Biopsies will be performed at 10 cm proximal to the malleoli. Punch biopsies and IENFD will not be performed in Cohort K.
- monitoring/recording of AEs/SAEs
- monitoring/recording of concomitant therapy

### Day 56 (±4 days): Follow-Up Visit

The following tests/assessments must be performed at the Day 56 (±4 days) Follow-Up Visit and should also be performed for subjects who withdraw from the study prematurely (4 weeks after the Premature Study Withdrawal Visit, if possible):

- physical examination
- vital signs (systolic and diastolic blood pressure, pulse, body temperature). The subject must remain in a resting position for 5 minutes prior to having his/her pulse and blood pressure taken.
- clinical neurological examination
- VAS of the SF‑MPQ
- Likert numerical pain rating assessment
- monitoring/recording of AEs/SAEs
- monitoring/recording of concomitant therapy

# Safety Definitions, Monitoring, and Reporting

Throughout the course of the study, every effort must be made to remain alert to possible adverse events (AEs). If an AE occurs, the first concern should be for the safety of the subject. If necessary, appropriate medical intervention should be provided.

At the signing of the ICF, each subject must be given the names and telephone numbers of study site staff for reporting AEs and medical emergencies.

## Definitions

### Serious Pre-Treatment Event

A serious pre‑treatment event is any event that meets the criteria for serious adverse event (SAE) reporting (as defined in Section 15.1.3) and occurs after the subject signs the ICF, but before administration of study treatment.

### Adverse Event

An AE is any untoward medical occurrence in a patient or clinical investigation subject administered a pharmaceutical product and that does not necessarily have a causal relationship with this treatment. An AE can therefore be any unfavorable and unintended sign (including an abnormal laboratory finding), symptom, or disease temporally associated with the use of a medicinal (investigational) product, whether or not related to the medicinal (investigational) product.

### Serious Adverse Event

An SAE is any untoward medical occurrence that at any dose:

- results in death
- in the view of the Investigator, places the subject at immediate risk of death (a life‑threatening event); however, this does not include an event that, had it occurred in a more severe form, might have caused death
- requires inpatient hospitalization or prolongation of existing hospitalization
- results in persistent or significant disability/incapacity
- results in a congenital anomaly/birth defect, or
- is a medically important event that, in the opinion of the Investigator, may jeopardize the subject or may require intervention to prevent one of the other outcomes listed in the definition above (Examples of such medical events include allergic bronchospasm requiring intensive treatment in an emergency room or convulsions occurring at home that do not require an inpatient hospitalization).

## Monitoring and Recording Events

### Serious Pre-Treatment Events

A serious pre-treatment event experienced by the subject after signing the ICF, but before administration of study treatment is to be recorded on the SAE Form and faxed to Biogen Idec Safety and Benefit-Risk Management (SABR) within 24 hours of the study site staff becoming aware of the event as described in Figure 15‑1.

### Adverse Events

Any AE experienced by the subject from the time of dosing with study treatment until the Day 56 Follow-Up Visit or Premature Study Withdrawal is to be recorded on the CRF, regardless of the severity of the event or its relationship to study treatment.

### Serious Adverse Events

Any SAE experienced by the subject from the time of dosing with study treatment until the Day 56 Follow-Up Visit or Premature Study Withdrawal is to be recorded on an SAE Form, regardless of the severity of the event or its relationship to study treatment. SAEs must be reported to the Sponsor (or designee) as detailed in Figure 15‑1.

Any SAE ongoing when the subject completes the study or discontinues from the study will be followed by the Investigator until the event has resolved, stabilized, or returned to baseline status.

### All Events

All events must be assessed to determine the following:

- If the event meets the criteria for an SAE as defined in Section 15.1.3.
- The relationship of the event to study treatment as defined in Section 15.3.1.
- The severity of the event as defined in Section 15.3.2.

### Immediate Reporting of Serious Adverse Events

In order to adhere to all applicable laws and regulations for reporting an SAE, the study site must formally notify Biogen Idec SABR within 24 hours of the study site staff becoming aware of the SAE. It is the Investigator’s responsibility to ensure that the SAE reporting information and procedures described in Figure 15‑1 are used and followed appropriately.

#### Death

The death must be recorded on the appropriate CRF. All causes of death must be reported as SAEs. The Investigator should make every effort to obtain and send death certificates and autopsy reports to Biogen Idec SABR or designee.

Figure 15-1 Reporting Information for SAEs

| Any Serious Event that occurs from the time that the subject has signed informed consent until the Day 56 Follow-Up Visit or Premature Study Withdrawal must be reported to Biogen Idec SABR within 24 hours of the study site staff becoming aware of the event.  A report ***must be submitted*** to Biogen Idec SABR regardless of the following:   - whether or not the subject has undergone study-related procedures - whether or not the subject has received study treatment - the severity of the event - the relationship of the event to study treatment   To report initial or follow‑up information on a Serious Event, fax a completed SAE form to the following:  Biogen Idec SABR  <fax # readacted> |
| --- |

## Safety Classifications

### Relationship of Events to Study Treatment

The following definitions should be considered when evaluating the relationship of AEs and SAEs to the study treatment:

| Relationship of Event to Investigational Drug | |
| --- | --- |
| Not related | An adverse event will be considered “not related” to the use of the investigational drug if there is not a possibility that the event has been caused by the product under investigation. Factors pointing toward this assessment include, but are not limited to: the lack of reasonable temporal relationship between administration of the drug and the event, the presence of a biologically implausible relationship between the product and the adverse event (e.g., the event occurred before administration of drug), or the presence of a more likely alternative explanation for the adverse event. |
| Related | An adverse event will be considered “related” to the use of the investigational drug if there is a possibility that the event may have been caused by the product under investigation. Factors that point toward this assessment include, but are not limited to: a positive re-challenge, a reasonable temporal sequence between administration of the drug and the event, a known response pattern of the suspected drug, improvement following discontinuation or dose reduction, a biologically plausible relationship between the drug and the adverse event, or a lack of an alternative explanation for the adverse event. |

### Severity of Events

The following definitions should be considered when evaluating the severity of AEs and SAEs:

| Severity of Event | |
| --- | --- |
| Mild | Symptom(s) barely noticeable to subject or does not make subject uncomfortable; does not influence performance or functioning; prescription drug not ordinarily needed for relief of symptom(s) but may be given because of personality of subject. |
| Moderate | Symptom(s) of a sufficient severity to make subject uncomfortable; performance of daily activity is influenced; subject is able to continue in study; treatment for symptom(s) may be needed. |
| Severe | Symptom(s) cause severe discomfort; symptoms cause incapacitation or significant impact on subject’s daily life; severity may cause cessation of treatment with study treatment; treatment for symptom(s) may be given and/or subject hospitalized. |

### Expectedness of Events

Expectedness of all AEs will be determined according to the IB.

## Prescheduled or Elective Procedures or Routinely Scheduled Treatments

A prescheduled or elective procedure or a routinely scheduled treatment will not be considered an SAE, even if the subject is hospitalized; the study site must document all of the following:

- The prescheduled or elective procedure or routinely scheduled treatment was scheduled (or was on a waiting list to be scheduled) prior to obtaining the subject’s consent to participate in the study.
- The condition requiring the prescheduled or elective procedure or routinely scheduled treatment was present before and did not worsen or progress between the subject’s consent to participate in the study and the time of the procedure or treatment.
- The prescheduled or elective procedure or routinely scheduled treatment is the sole reason for the intervention or hospital admission.

## Procedures for Handling Special Situations

### Overdose

An overdose is any dose of study treatment given to a subject or taken by a subject that exceeds the dose described in the protocol. Overdoses are not considered AEs, however, all overdoses should be recorded on an SAE Form and faxed to Biogen Idec within 24 hours. An overdose should be recorded even if it does not result in an AE. Overdoses do not need to be recorded in the CRF.

### Medical Emergency

In a medical emergency requiring immediate attention, study site personnel will apply appropriate medical intervention, according to current standards of care, and contact the <name and phone numbers readacted>

### Contraception Requirements

All male subjects and female subjects of child-bearing potential must practice effective contraception during the study and be willing and able to continue contraception for 2 months after their last dose of study treatment.

For purposes of this study, effective contraception is defined as follows:

For females:

- Using 1 or more of the following acceptable methods of contraception: surgical sterilization (i.e., no uterus and/or ovaries), intrauterine contraception/device, hormonal contraception, or any 2 barrier methods (a combination of male or female condom with spermicide; diaphragm, sponge, cervical cap).
- Abstinence can be considered an acceptable method of contraception at the discretion of the Investigator. Periodic abstinence (e.g., calendar, ovulation, symptothermal, post‑ovulation methods) and withdrawal are not considered acceptable methods of contraception.

For purposes of this study, women of child-bearing potential (WOCBP) are defined as all women physiologically capable of becoming pregnant, **UNLESS** they meet the following conditions:

- Post-menopausal: 12 months of natural (spontaneous) amenorrhea or 6 weeks post surgical bilateral oophorectomy.
- Hysterectomy.

For males:

- Effective male contraception includes a vasectomy with negative semen analysis at follow‑up, or the use of condoms with spermicide.

### Pregnancy

**Subjects should not become pregnant.**

The Investigator must report the pregnancy by faxing the appropriate form to Biogen Idec SABR within 24 hours of the study site staff becoming aware of the pregnancy, at <fax # readacted>The Investigator or study site staff must report the outcome of the pregnancy to Biogen Idec SABR.

Please note that congenital abnormalities/birth defects in the offspring of male of female subjects when conception occurred during study treatment administration should be reported.

### Unblinding for Medical Emergencies

In this study, treatment assignment information is held by the Pharmacist. For medical emergencies, the Investigator will be provided with a set of numbered and sealed code‑break envelopes that will contain the treatment assignment for each enrolled subject.

All treatment assignment information, including code‑break envelopes, must be kept in a secure location.

In a medical emergency when knowledge of the subject’s treatment assignment may possibly influence the subject’s clinical care, the Investigator or designee should attempt to contact the Biogen Idec Medical Director at the contact numbers provided in Section 15.5.2 to discuss the emergency whenever possible. In these instances, the Investigator or designee may open the code‑break envelope that corresponds to the subject experiencing the event. The date and the reasons for opening the code‑break envelope must be submitted to Biogen Idec within 24 hours of the unblinding. Any opened envelopes must be returned to the secure location. Code‑break envelopes will be checked at monitoring visits and will be collected at the end of the study.

The Investigator must document the reasons for unblinding in the subject’s source documents. The Investigator is strongly advised not to divulge the subject’s treatment assignment to any individual not directly involved in managing the medical emergency nor to personnel involved with the analysis and conduct of the study.

### Regulatory Reporting

Suspected Unexpected Serious Adverse Reactions (SUSARs) are SAEs that are unexpected and judged by the Investigator or the Sponsor to be related to the study treatment administered.

SUSARs will be unblinded for reporting to the appropriate authorities and central ethics committees by appropriate personnel in Biogen Idec SABR (or designee). A blinded report will be sent to the Investigator. Personnel involved in the analysis of the study will also remain blinded. The subject may remain in the study and continue to receive study treatment at the Investigator’s discretion. The Investigator should assume that the subject received active treatment when making this decision.

## Investigator Responsibilities

The Investigator’s responsibilities include the following:

- Monitor and record all AEs, including SAEs, regardless of the severity or relationship to study treatment.
- Determine the seriousness, relationship, and severity of each event.
- Determine the onset and resolution dates of each event.
- Monitor and record all pregnancies and follow-up on the outcome of the pregnancy.
- Complete an SAE form for each SAE and fax it to Biogen Idec SABR within 24 hours of the study site staff becoming aware of the event.
- Pursue SAE follow‑up information actively and persistently. Follow‑up information must be reported to Biogen Idec SABR within 24 hours of the study site staff becoming aware of new information.
- Ensure all AE and SAE reports are supported by documentation in the subjects’ medical records.
- Report SAEs to local ethics committees, as required by local law.

## Biogen Idec Responsibilities

Biogen Idec’s responsibilities include the following:

- Before study site activation and subject enrollment, the Clinical Monitor is responsible for reviewing with study site staff the definitions of AE and SAE, as well as the instructions for monitoring, recording, and reporting AEs and SAEs.
- Biogen Idec is to notify all appropriate regulatory authorities, central ethics committees, and the Investigator of SAEs, as required by local law, within required time frames.

# Statistical Statement and Analytical Plan

## Description of Objectives and Endpoints

### Primary Objective and Endpoints

The primary objective of the study is to determine the safety/tolerability profile, systemic PK behavior, and immunogenicity of single IV and SC administrations of BG00010 to sciatica subjects.

The primary safety/tolerability/immunogenicity endpoints are:

- The number and proportion of subjects with AEs.
- Assessment of clinical laboratory parameters.
- Assessment of vital signs.
- Assessment of pain as measured by a Likert numerical pain rating scale.
- Longitudinal assessment of QST (Vibratory, Cool Thermal, and Heat Pain thresholds).
- Assessment of IENFD.

The PK parameters anticipated to be calculated and reported include:

- C_max_: maximum observed serum concentration
- AUC: area under the serum concentration curve
- t_½_: terminal half-life
- CL: total body clearance
- V_ss_: steady state volume of distribution

## Demography

All appropriate background data will be summarized by presenting frequency distributions and/or basic summary statistics (mean, standard deviation [SD], median, and range).

## Pharmacokinetics

### Analysis Population

The PK analysis population is defined as all subjects who are randomized and who have been dosed with study treatment and have measurable BG00010 concentrations from at least 1 collected sample.

### Methods of Analysis

PK parameters will be summarized by dose cohort using descriptive statistics to provide an initial assessment of the PK properties of BG00010. PK parameters will be generated for each subject using the concentration versus time data obtained from measurement of BG00010 in blood samples. A validated computer program such as WinNonlin 5.0, or equivalent, will be used to process the data. PK parameters to be calculated will include, but will not be limited to, those noted in Section 16.1.1.

## Safety Data

### Analysis Population

The safety population is defined as all subjects who are randomized and have been dosed with study treatment.

### Methods of Analysis

Adverse Events

The incidence of treatment-emergent AEs and SAEs will be summarized for each dosing cohort overall, by severity, and by relationship to study treatment. The summary tables will include incidence estimates for system organ classes as well as for individual preferred term within each system organ class. A treatment-emergent AE is defined as any AE that has onset on or after dosing with study treatment, or any pre-existing condition that has worsened after dosing with study treatment. SAEs and AEs resulting in withdrawal from the study will be summarized by cohort. AEs will be coded using the Medical Dictionary for Regulatory Activities (MedDRA).

Laboratory Evaluations

Laboratory results that are outside of the normal range for each parameter will be identified and evaluated for their clinical relevance.

Vital Signs

Vital signs will be descriptively examined to determine the incidence of clinically relevant abnormalities, and summarized by cohort.

ECG

A listing of subjects with abnormal ECG status will be presented. Changes from baseline will be summarized by cohort.

Pain Severity Score (Likert)

Change from baseline to endpoint in the severity score from an 11-point Likert scale will be analyzed.

QST

Change from baseline to endpoint in QST will be analyzed. Results will be summarized by cohort and compared with the respective placebo group. Although data from healthy volunteers will not be collected as part of the study, the results from the study will be compared to this background reference population (from published literature) to determine if there are any significant deviations.

IENFD

Change from baseline to endpoint in IENFD will be analyzed. Changes will be summarized by cohort and compared with the respective placebo group as a background reference population.

Physical Examination

A listing of subjects with normal status at baseline, but abnormal status at any time after the date of dosing will be presented.

Clinical Neurological Examination

A listing of subjects with normal status at baseline, but abnormal status at any time after the date of dosing will be presented.

## Immunogenicity Data

### Analysis Population

The analysis population for immunogenicity is defined as all subjects who are randomized and who have been dosed with study treatment and have immunogenicity data collected postdosing.

### Methods of Analysis

Immunogenicity will be summarized by cohort using descriptive statistics to provide an initial assessment of the immunogenic properties of BG00010.

## Data Analysis for Additional Assessments

Exploratory analyses of targeted biomarkers will be performed to identify biomarkers that may be indicative of BG00010 activity.

Change from baseline in biomarker analyses parameters will be summarized by dose.

If transcript profiling (whole blood) and/or global proteomics analysis (plasma) are performed, the assessment will be treated as a separate investigational report.

## <interim analysis redcated>

## Sample Size Considerations

The sample size calculation is based on the design of a classical Phase 1 study and is not based on any study power consideration.

# Ethical Requirements

Biogen Idec and the Investigator must comply with all instructions, regulations, and agreements in this protocol and applicable International Conference on Harmonisation (ICH) and Good Clinical Practice (GCP) guidelines and conduct the study according to local regulations.

## Declaration of Helsinki

The Investigator must follow the recommendations contained in the Declaration of Helsinki, amended at the 52nd General Assembly in Edinburgh, Scotland, dated October 2000, with Notes of Clarification in 2002 (Washington) and 2004 (Tokyo).

## Ethics Committee

The Investigator must obtain ethics committee approval of the protocol, ICF, and other required study documents prior to starting the study.

If the Investigator makes any changes to the ICF, Biogen Idec must approve the changes before the ICF is submitted to the ethics committee. A copy of the approved ICF must be provided to Biogen Idec. After approval, the ICF must not be altered without the agreement of the relevant ethics committee and Biogen Idec.

It is the responsibility of the Investigator to ensure that all aspects of institutional review are conducted in accordance with current governmental regulations.

Biogen Idec must receive a letter documenting ethics committee approval, which specifically identifies the protocol, protocol number, and ICF, prior to the initiation of the study. Protocol amendments will be subject to the same requirements as the original protocol.

A progress report must be submitted to the ethics committee at required intervals and not less than annually.

At the completion or termination of the study, the study site must submit a close‑out letter to the ethics committee and Biogen Idec.

## Subject Information and Consent

Prior to any testing under this protocol, including screening tests and assessments, written informed consent with the approved ICF must be obtained from the subject in accordance with local practice and regulations. Written informed consent must be obtained from all subjects participating in a clinical study conducted by Biogen Idec.

The background of the proposed study, the procedures, and the benefits and risks of the study must be explained to the subject. The subject must be given sufficient time to consider whether to participate in the study.

A copy of the ICF, signed and dated by the subject, must be given to the subject. Confirmation of a subject’s informed consent must also be documented in the subject’s medical record prior to any testing under this protocol, including screening tests and assessments.

Each consent form should contain an authorization allowing the Principal Investigator(s) and Biogen Idec to use and disclose PHI (i.e., subject-identifiable health information) in compliance with local law.

The signed consent form will be retained with the study records.

## Subject Data Protection

Prior to any testing under this protocol, including screening tests and assessments, candidates must also provide all authorizations required by local law (e.g., PHI authorization in North America).

The subject will not be identified by name in the CRF or in any study reports, and these reports will be used for research purposes only. Biogen Idec, its partner(s) and designee(s), and various government health agencies may inspect the records of this study. Every effort will be made to keep the subject’s personal medical data confidential.

# Administrative Procedures

## Study Site Initiation

The Investigator must not screen any subjects prior to completion of a study initiation visit, conducted by Biogen Idec or designee. This initiation visit will include a detailed review of the protocol and study procedures.

## Quality Assurance

During and/or after completion of the study, quality assurance officers named by Biogen Idec or the regulatory authorities may wish to perform on‑site audits. The Investigator will be expected to cooperate with any audit and to provide assistance and documentation (including source data) as requested.

## Monitoring of the Study

The Principal Investigator(s) must permit study-related monitoring by providing direct access to source data and to the subjects’ medical histories.

The Clinical Monitor(s) will visit the Investigator(s) at regular intervals during the course of the study and, as appropriate, after the study has completed.

During these visits, CRFs and supporting documentation related to the study will be reviewed and any discrepancies or omissions will be resolved.

The monitoring visits must be conducted according to the applicable ICH and GCP guidelines to ensure protocol adherence, quality of data, study treatment accountability, compliance with regulatory requirements, and continued adequacy of the study site and its facilities.

## Study Funding

All financial details are provided in the separate contract between the institution and Biogen Idec.

# Further Requirements and General Information

### Data Coordinating Center

Biogen Idec will be responsible for all administrative aspects of this study including, but not limited to, study initiation, monitoring, management of AEs, and data management.

### Central Laboratories for Laboratory Assessments

Testing for all urinalysis, urine drug screen, pregnancy, hepatitis/HIV, hematology, and blood chemistry samples will be performed locally. IENFD, PK, and antibody samples will be tested at qualified analytical testing laboratories. Biomarker samples will be tested at a qualified analytical testing laboratory.

## Drug Safety Review Committee

A Drug Safety Review Committee (DSRC) will be formed to review unblinded safety data.

Prior to escalation to the next planned dose level, the DSRC will review unblinded safety data (AEs; all SAEs; vital signs; results for laboratory tests, Likert numerical pain rating assessments, QST, and IENFD) through Day 28, and PK data through Day 5 (or an earlier time point if the concentration drops below the limit of quantitation prior to Day 5) from all subjects in the preceding cohorts, to determine dose tolerability and systemic exposure to BG00010. Enrollment of the next cohort (the next planned dose level) will not begin until the DSRC has approved dosing for that cohort.

Dosing will be suspended if a subject experiences a treatment-related increase of 4 points from baseline in one of their pain evaluations, a treatment-related SAE, or a treatment-related Grade 3 or higher laboratory AE during dosing of a cohort. The DSRC will complete a safety evaluation (including assessment of the nature and severity of the SAE or laboratory AE, if applicable) and decide whether or not to continue dosing the cohort.

Members of the DSRC will include a Biogen Idec Medical Director (or designee), a Biogen Idec Statistician (or designee), a Biogen Idec Drug Safety Representative (or designee), the Investigator, and an external independent person.

## Changes to Final Study Protocol

All protocol amendments must be submitted to the ethics committee. Protocol modifications that affect subject safety, the scope of the investigation, or the scientific quality of the study must be approved by the ethics committee before implementation of such modifications to the conduct of the study. If required by local law, such modifications must also be approved by the appropriate regulatory agency prior to implementation.

However, Biogen Idec may, at any time, amend this protocol to eliminate an apparent immediate hazard to a subject. In this case, the appropriate regulatory authorities will be notified subsequent to the modification.

In the event of a protocol modification, the subject consent form may require similar modifications (see Sections 17.2 and 17.3).

## Ethics Committee Notification of Study Completion or Termination

Where required, the Health Authorities and ethics committees must be notified of completion or termination of this study, and sent a copy of the study synopsis in accordance with necessary timelines.

## Retention of Study Data

The Principal Investigator must maintain all Essential Documents (as listed in the ICH Guideline for GCP) until notified by Biogen Idec and in accordance with all local laws regarding retention of records.

## Study Report Signatory

The Investigator will be a signatory for the study report.

# References

Baloh R, Tansey M, et al. Artemin, a novel member of the GDNF ligand family, supports peripheral and central neurons and signals through the GFRalpha3-RET receptor complex. Neuron 1998;21(6):1291-1302.

Bolon B, Jing S, Asuncion F, et al. The candidate neuroprotective agent artemin induces autonomic neural dysplasia without preventing peripheral nerve dysfunction. Toxicol Pathol 2004;32:275-294.

Common Terminology Criteria for Adverse Events, Version 5.0, Cancer Therapy Evaluation Program. http:/ctep.cancer.gov. August 9, 2006.

Frymoyer, J. Back pain and sciatica. N Engl J Med 1988;318(5):291-300.

Frymoyer, J. Lumbar disk disease: epidemiology. Instr Course Lect 1992; 41: 217-23.

Koes, B, van Tulder W, et al. Diagnosis and treatment of sciatica. Br Med J 2007;334(7607): 1313-7.

Likert R. A technique for the development of attitude scales for multi-term assessments of the same variable. Educational and Psychological Measurement 1952;12:313-315.

Malin S, Molliver D, Koerber H, et al. GDNF family members sensitize nociceptors in vitro and produce thermal hyperalgesia in vivo. J Neurosci 2006;26:8588-8599.

Neublastin (BG00010) Investigator's Brochure, Biogen Idec Inc., 2007.

Porreca F, Ossipov M, Gebhart G. Chronic pain and medullary descending facilitation. Trends Neurosci 2002;25:319-25.

Weber H., Holme I, et al. The natural course of acute sciatica with nerve root symptoms in a double-blind placebo-controlled trial evaluating the effect of piroxicam. Spine 1993; 18(11): 1433-8.

# Signed agreement of the study protocol

I have read the foregoing protocol, “A Single-Center, Randomized, Blinded, Placebo‑Controlled, Single-Administration, Sequential‑Cohort, Dose-Escalation Study to Evaluate the Safety, Tolerability, and Pharmacokinetics of BG00010 (Neublastin) Administered to Sciatica Subjects,” Version 7, and agree to conduct the study according to the protocol and the applicable ICH guidelines and GCP regulations, and to inform all who assist me in the conduct of this study of their responsibilities and obligations.

____________________________________________________

Investigator’s Signature Date

____________________________________________________

Investigator’s Name (Print)

____________________________________________________

Study Site (Print)
